# Nano‐bio‐interface: Unleashing the Potential of Noble Nanometals

**DOI:** 10.1002/smsc.202300227

**Published:** 2024-02-05

**Authors:** Peng Guo, Yiyun Wang, Hongbo Cui, Xiang Yao, Guijian Guan, Ming‐Yong Han

**Affiliations:** ^1^ Institute of Molecular Plus Tianjin University 92 Weijin Road Tianjin 300072 China

**Keywords:** bioapplications, functionality, nano‐bio‐interface, noble nanomaterials

## Abstract

Engineered noble metal nanomaterials (NMN) possess adjustable optical, electrical, and biocompatible properties that make them excellent tools for probing the nano‐bio‐interface. Understanding their interactions with biomolecules, cells, and tissues at the nano‐bio‐interface is crucial in designing these nanomaterials for biomedical applications. This review summarizes the structure, properties, synthesis, and passivation methods of noble metal nanoparticles, as well as the construction strategy and detection technology of the nano‐bio‐interface to provide important information about their uptake, distribution, metabolism, and degradation in vivo and in vitro. The related action mechanisms include the kinetic and thermodynamic processes of the nano‐bio‐interface, the driving forces for its formation, and the chemical reactions at the nano‐bio‐interface. By exploring the action mechanism of the nano‐bio‐interface, the antibacterial properties and cytotoxicity of NMN could be better understood, and open up more extensive biological applications. Finally, the future trends of NMNs in the biological field and the challenges encountered in realizing these technologies are discussed.

## Graphical abstract

Engineered noble metal nanomaterials (NMNs) possess adjustable optical, electrical, and biocompatible properties that make them excellent tools for probing the nano‐bio‐interface. Understanding their interactions with biomolecules, cells, and tissues at the nano‐bio‐interface is crucial in designing these nanomaterials for biomedical applications. This review aims to investigate the factors that influence the interactions, and explore the underlying mechanisms of their biological applications.

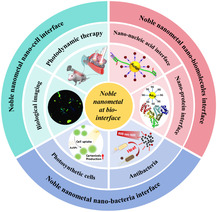

## 1 Introduction

Nano‐bio‐interfaces explore the convergence of subcellular biomolecular mechanisms operating at the nanoscale and their interactions with nanoscale materials. The studies of fundamental nano‐bio‐interfaces help us understand and predict how nanomaterials behave in a biological environment and how biomolecules respond to nanomaterials. Moreover, it has inspired attempts to artificially replicate biologically responsive stimuli, enabling the modulation of biological processes. During the formation of nano‐bio‐interfaces, various driving forces come into play. These forces not only cause biomolecules to undergo re‐conformation but also affect the surface structure and function of the nanomaterials themselves. However, the formation of nano‐bio‐interface also has positive effects, such as mitigating the biotoxicity of nanomaterials, thereby enhancing their potential for biological applications. Nevertheless, several challenges need to be overcome for a comprehensive understanding and applications of nano‐bio‐interface theory at a higher level. The composition and structure of nano‐bio‐interface can be easily influenced by the characteristics of nanomaterials. Even slight differences in size can lead to distinct biological outcomes. Additionally, the complexity of the micro‐environment (including the variety of biomolecules and properties of the biological medium) poses further challenges in studying nano‐bio‐interface.

Noble metal nanomaterials (NMNs) with highly chemical stability are extensively utilized in biologically‐related applications due to their remarkable optical, electrical, catalytic, and biological stability, while many other non‐noble metal nanomaterials such as silica and polymers can serve as surface‐coating materials to improving their surface functionality and biocompatibility.^[^ ^1^ ^]^ In contrast, metal oxide nanomaterials can result in the release and diffusion of metal ions into surrounding tissues, potentially leading to metal‐related diseases. Polymer materials, in contrast, are prone to degradation within the body, consequently releasing toxic and carcinogenic small molecules.^[^ ^2^ ^]^ Different fabrication methods, such as top‐down (grinding, vapor deposition, laser ablation, and pyrolysis) and bottom‐up (chemical, photochemical, electrochemical, hydrolytic, and biological) approaches, can be employed to produce NMNs.^[^ ^3^ ^]^ Achieving uniformity in size, shape, and surface characteristics of NMNs is crucial for their effective application in biomedicine, biological imaging, diagnosis, and therapy.^[^ ^4^ ^]^ By leveraging nanotechnology principles and techniques to bind NMNs with biomolecules, advanced biotechnological processes can be enhanced, developed, and better understood. Several methods such as near‐infrared imaging, electrochemical sensing, surface‐enhanced Raman spectroscopy, colorimetric, and fluorescence methods have been utilized in the biomedical analysis of NMNs. Each method has its own advantages and disadvantages when applied to real sample analysis in biomedical applications. The rapid progress of nanotechnology has significantly contributed to the utilization of NMNs in biomedicine. This progress has presented opportunities for the early development of novel diagnostic platforms for disease detection and threat identification.

**Figure 1:**
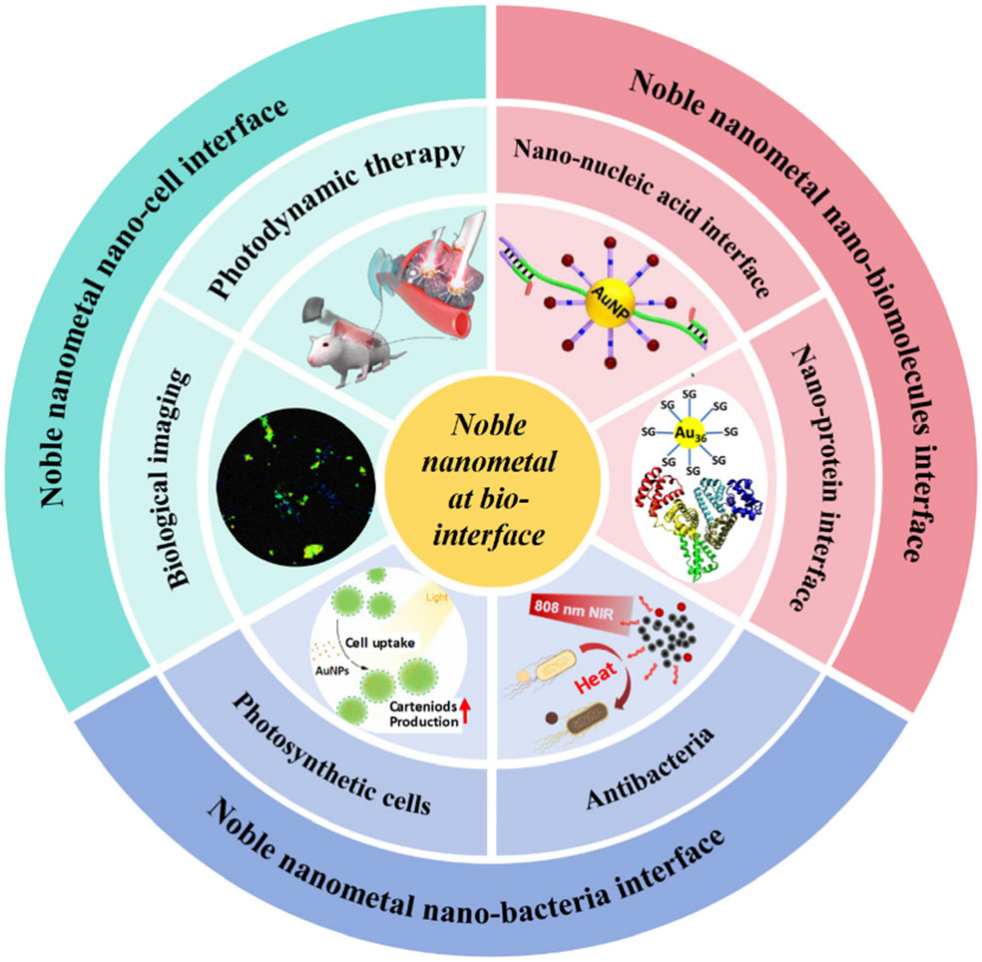
Noble metal nanomaterials at the bio‐interface. Adapted with permission.^[^ ^165^ ^]^ Copyright 2023, American Chemical Society. Adapted with permission.^[^ ^166^ ^]^ Copyright 2017, American Chemical Society. Adapted with permission.^[^ ^167^ ^]^ Copyright 2022, American Chemical Society. Adapted with permission.^[1h]^ Copyright 2020, American Chemical Society. Adapted with permission.^[^ ^168^ ^]^ Copyright 2023, American Chemical Society. Adapted with permission.^[^ ^169^ ^]^ Copyright 2023, American Chemical Society.

This review aims to investigate the factors that influence the interactions between NMNs and biological components at the nano‐bio‐interface, and to explore the underlying mechanisms of their biological applications (**Figure** **1**). Firstly, it focuses on the structure, properties, and fabrication methods of NMNs, as well as the strategies for constructing the nano‐bio‐interface using NMNs. Secondly, it reviews the thermodynamic and kinetic processes involved in the formation of nano‐bio‐interfaces and the interaction forces that come into play. This section also examines chemical reactions that occur at the nano‐bio‐interface, such as the generation and elimination of reactive oxygen species (ROS) and the presence of surface defects on NMNs. Thirdly, it highlights various techniques used to probe the nano‐bio‐interface, providing an extensive literature survey of recent advances in this field. Finally, the review delves into the biotoxicity of NMNs in practical applications and focuses on the factors that influence their biotoxicity. The objectives of this article are threefold: 1) To elucidate the intriguing properties associated with the nano‐bio‐interface of NMNs; 2) To provide a brief overview of the NMNs in advancing nanobiomedicine; and 3) To assess the potential of NMNs as functional components in biomedical applications.

## 2 Noble Metal Nanomaterials

**Figure 2:**
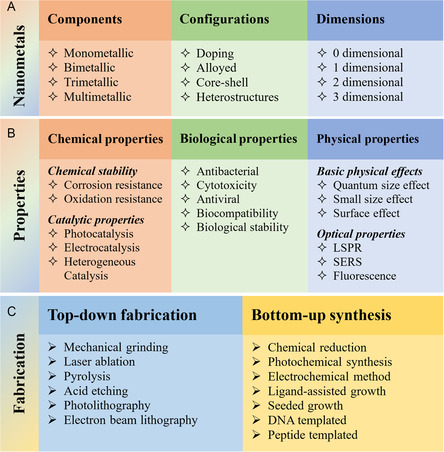
Noble metal nanomaterials showcasing (A) diverse components, configurations, and dimensions, (B) their corresponding properties, and (C) various fabrication/synthesis methods used.

There are eight noble metals in order of atomic number, including rhodium (Rh), ruthenium (Ru), palladium (Pd), silver (Ag), osmium (Os), iridium (Ir), platinum (Pt), and gold (Au).^[^ ^5^ ^]^ The corresponding NMNs possess similar properties to pure noble metals, endowing unique physical and chemical properties (such as optical, electrical, magnetic, optoelectronic, thermal, catalytic, and biological properties), and rendering them with broad application prospects in various fields such as high‐efficiency catalysis,^[^ ^6^ ^]^ environmental protection,^[^ ^7^ ^]^ and biomedical science (**Figure** **2**).[^1^, ^8^] As of now, there exists a wide array of methods for preparing NMNs. Through the utilization of diverse preparation techniques, we have nearly attained precise control over the size, shape, morphology, surface charge, and other inherent properties of NMNs. These physical properties play a pivotal role in determining the practical applications of NMNs. The selection of the preparation method for NMNs directly influences their surface properties, while the physical attributes dictate the types of nano‐bio‐interfaces they can form. Ultimately, these factors collectively determine the fate of NMNs in practical applications.

### 2.1 Multi‐componential Nanostructures

In general, the NMNs can be divided into single and multi‐componential NMNs compositionally (Figure 2A). Single‐componential NMNs include nanoparticles, nanowires, and nanocubes of gold, silver, palladium, platinum, rhodium, iridium, ruthenium, and osmium nanoparticles (i.e., AgNPs, AuNPs, PdNPs, PtNPs, RhNPs, IrNPs, RuNPs, and OsNPs) with sizes ranging from 1 to 100 nm.^[^ ^9^ ^]^ Among them, Au and Ag nanomaterials have been extensively explored due to their well‐established synthetic methods, tunable properties, biocompatibility, and safety. Especially, Au nanorods, nanocages, and core–shell nanoparticles^[^ ^10^ ^]^ have found applications in bioimaging, biodiagnostic assays, drug delivery, and photothermal therapy.

#### 2.1.1 Multi‐components

In parallel, multi‐componential NMNs are prepared by mixing two or more noble metals in solid solutions, resulting in diverse nanostructures such as binary (e.g., AuAg,^[^ ^11^ ^]^AgPt,^[^ ^12^ ^]^ AuPt,^[^ ^13^ ^]^ AuPd,^[^ ^14^ ^]^and PdRu^[^ ^15^ ^]^), ternary (e.g., AuAgPd,^[^ ^16^ ^]^ AuPtAg,^[5d]^ AuPtRu,^[^ ^17^ ^]^ and AuPtPd^[^ ^18^ ^]^) and multinary (e.g., PtPdRhRuCu) systems.^[5e]^ The performance and application of these NMNs are greatly influenced by their size, shape, morphology, and composition. Besides sizes, various shapes, including nanospheres, nanorods, nanowires, and nanocubes, can be readily designed according to specific requirements. Additionally, the internal distribution of elements within the NMNs also affects their overall performance.^[3a]^

#### 2.1.2 Dimensionality

The NMNs exhibit unique effects due to their small size in one or more spatial dimensions. These effects include size, surface, quantum size, and macroscopic quantum tunneling effects. The NMNs can be prepared in various dimensional structures (Figure 2A), including 0D (e.g., nanoclusters and nanoparticles), 1D (e.g., nanowires, nanotubes, and nanorods), 2D structures (e.g., nanoplates, nanosheets, nanowheels, and nanohelices), and 3D structures inside (e.g., nanocages, nanorings, nanoframes and nanostars). Various NMNs have been exploited, including wire‐ and honeycomb‐like assemblies of OsNPs,^[^ ^19^ ^]^ protein‐Au nanoclusters achieving efficient intracellular colocalization, uptake and transfection,^[10a]^ highly emissive and water‐soluble Ag nanoclusters,^[^ ^20^ ^]^ diameter‐controlled and single‐crystal Au nanospheres with excellent uniformity,^[9e]^ and uniform Ir nanoparticles with superior catalytic activity and stability in the hydrogen evolution reaction.^[9d]^ Other synthesized NMNs include highly anisotropic Ag and Pt nanorods/nanowires,^[^ ^21^ ^]^ vertically‐aligned bimetallic Pt/Pd nanotube arrays for higher catalytic activity in methanol oxidation,^[^ ^22^ ^]^ and gold nanorods stabilized by grafting with thermoresponsive block copolymers with potential applications in drug delivery and photothermal therapy.^[10b]^

In recent years, there has been extensive attention on 2D and 3D NMNs. Ultrathin Pt nanoplates showed significant improvements in specific activity, mass activity, and catalytic stability compared to commercial Pt/C catalyst.^[^ ^23^ ^]^ Ru nanosheets exhibited excellent catalytic activity and were used in developing a sensor array for protein recognition.^[10c]^ Pt nanowheels and Pt nanohelices with enhanced properties were synthesized using specific assembly techniques.^[^ ^24^ ^]^ Au‐Ag nanocages with well‐defined pores were formed by reacting Ag nanocubes with HAuCl_4_. Pt@Au nanorings were created by growing Pt on the edge of Au nanoplates, enhancing structural stability.^[^ ^25^ ^]^ Ultrathin octahedral Au_3_Ag nanoframes exhibited high electrocatalytic activity in methanol oxidation.^[^ ^26^ ^]^ Gold nanostars were prepared using a simple and eco‐friendly synthesis method.^[^ ^27^ ^]^

### 2.2 Physical and Chemical Properties

NMNs have garnered considerable attention owing to their unique optical properties that differ from bulk materials (Figure 2B). In addition, their surface stabilization and passivation further can alter their surface properties to improve biocompatibility for applications.

#### 2.2.1 Optical Properties

NMNs exhibit distinct characteristics such as localized surface plasmon resonance (LSPR) and surface‐enhanced Raman scattering (SERS). LSPR occurs when noble metal nanoparticles with sizes ranging from 3 to 100 nm coupled with incident light of wavelengths between 400 and 900 nm. This interaction leads to the collective oscillation of free electrons on the nanometal surface, arising from the coupling between the incident light's oscillating electric field and the conduction electrons of the nanoparticles. Consequently, metal nanoparticles strongly absorb photons that match their vibration frequency when LSPR occurs.^[^ ^28^ ^]^ The LSPR absorption peaks of different noble nanometals, such as gold nanoparticles (AuNPs) and silver nanoparticles (AgNPs), are mainly located in the visible and near‐infrared regions, while those of palladium, platinum, ruthenium, and rhodium nanoparticles (i.e., PdNPs, PtNPs, RuNPs, and RhNPs) are situated in the ultraviolet region. The absorption characteristics of LSPR are influenced by various factors, including nanoparticle size, morphology, structure, and the refractive index of the surrounding medium. By adjusting the size of nanoparticles, the LSPR absorption peak can be tuned accordingly. For example, increasing the aspect ratio of Au nanorods leads to a red shift in the longitudinal LSPR absorption peak. Moreover, nanoparticles of different sizes exhibit diverse scattering and absorption characteristics, resulting in varied colors observed in colloidal solutions. The symmetry of nanoparticles also affects the number and intensity of LSPR absorption peaks. Additionally, by controlling the morphology and composition of noble metal nanoparticles with structures like nanorods, nanoprisms, nanostars, and core–shells, the LSPR absorption peaks can be tuned from the visible to the near‐infrared region. Furthermore, different metal atoms possess distinctive inherent optical constants and charge densities, resulting in variations in their LSPR absorption peaks. For instance, 20 nm AuNPs appear wine‐red with an LSPR absorption peak around 520 nm, while AgNPs of the same size appear dark green with an LSPR absorption peak around 390 nm. However, in Au‐Ag alloy structures, the absorption band range is shifted to around 400–450 nm.

Apart from LSPR, noble metal nanoparticles have been found to either quench or enhance the fluorescence intensity of fluorophores.^[^ ^29^ ^]^ When the size of fluorescent metal nanoclusters approaches the Fermi wavelength of electrons, the continuous state density is transformed into discrete energy levels, exhibiting molecule‐like properties such as large Stokes shift and tunable luminescence. Consequently, NMNs hold significant potential in areas such as analysis, detection, and biological imaging. He et al. observed that AuNPs synthesized through the citrate reduction of chloroauric acid with sizes ranging in the tens of nanometers exhibited certain fluorescence properties.^[^ ^30^ ^]^ These nanoparticles showed a narrow full width at half‐maximum of 17 nm, and as particle sizes increased, the emission intensity gradually increased while the emission wavelength remained almost constant at 610 nm. Furthermore, the use of AuNPs as fluorescent probes for imaging HeLa cells resulted in high‐quality cell images.

#### 2.2.2 Surface Stabilization and Passivation

It is very important to stabilize and passivate the surface of NMNs role in their synthesis and application. The strong surface interaction energy between nanoparticles makes them prone to agglomeration. Therefore, in the process of synthesizing NMNs during the preparation process, a protective agent or stabilizer with dispersing properties is often employed to coat the surface of metal nanoparticles. This coating helps restrict the growth of nanoparticles, control their size and morphology, and stabilize the nanostructures against aggregation.^[6b]^ Various passivation approaches have been developed into four categories to enhance the stability and functionality of NMNs for expanding applications in biocompatible, sensing, and targeted systems. First, forming alloy nanostructures allows for improved stability by incorporating additional elements into the noble metal lattice. Second, fabricating core–shell nanostructures involves coating the noble metal core with functional shells, enhancing stability, and enabling surface functionalization. Third, utilizing organic ligands as surface coatings to stabilize NMNs, providing steric hindrance and electrostatic repulsion to prevent agglomeration. Finally, employing biological molecules as surface coatings, offering stability through specific molecular interactions.

Alloy nanostructures have been extensively studied to achieve high performance and stability in NMNs. For instance, the formation of alloys by combining different metals, such as Ag and Au, has been explored to maintain compositional homogeneity and enhance stability. Gao et al. conducted a surface‐protected annealing process to produce fully alloyed Ag/Au nanospheres,^[^ ^31^ ^]^ produced effectively stabilizing the Ag nanoparticles. By combining the excellent plasmonic properties of Ag with the remarkable stability of Au, Ag/Au alloy nanoparticles exhibited strong LSPRs with narrow bandwidths and high resistance to corrosive environments. An effective approach for stabilizing noble metal nanostructures is the use of silica coating to prepare functional core–shell nanoparticles. By applying a coating of inorganic materials, such as silica or titanium dioxide, onto the noble metal core, the resulting core–shell structure can provide stability and prevent the agglomeration and sintering of the noble metal under harsh conditions. This coating technique ensures the integrity and activity of the noble metal nanoparticles, making it a valuable method for numerous applications. For instance, Zhang et al. utilized the sol–gel method to transform silica particles into porous shells and hollow spheres,^[^ ^32^ ^]^ obtaining Au@SiO_2_ core–shell structures with AuNPs embedded in the silica shell. The porous silica shell provided excellent stabilization of AuNPs, preventing aggregation and maintaining their activity. Even after multiple reaction cycles, the optical absorption and morphology of AuNPs remained almost unchanged, underscoring the effectiveness of the silica shell for stabilizing the gold nanoparticles.

Various organic ligands, including poly(vinyl pyrrolidone) (PVP),^[^ ^33^ ^]^ polyvinyl alcohol (PVA),^[^ ^34^ ^]^ polyethylene glycol (PEG),^[^ ^35^ ^]^ cetyltrimethylammonium bromide (CTAB)^[^ ^36^ ^]^ and sodium dodecyl sulfate (SDS),^[^ ^37^ ^]^ can be used to stabilize noble metal nanostructures. The stabilization of these structures via organic ligands is typically achieved through electrostatic and steric repulsion. This approach not only helps control the size and shape of noble metal nanostructures but also prevents them from aggregating. However, the surface passivation of by organic ligands can block active sites on the metal surface and prevent analytes from attaching to the metal surface, resulting in weak Raman signals and low detecting sensitivity. Biomolecules, including peptides, deoxyribonucleic acid (DNA), and proteins, offer interesting options as surface coatings for NMNs due to their aqueous dispersibility and biocompatibility. These molecules may possess different metal binding sites and surface functional groups appropriate for biological purposes. For instance, during the preparation of bovine serum albumin (BSA)‐mediated Au nanoplates, proteins are considered to be preferentially adsorbed on the (111) facets at low pH, inhibiting growth in the (111) direction and resulting in the protein‐directed formation of anisotropic nanoplates.^[^ ^38^ ^]^

### 2.3 Fabrication and Synthesis

NMNs can be prepared using both top‐down and bottom‐up approaches. In the top‐down approach (Figure 2C), block‐like materials are reduced to nanoscale particles using various physical or chemical methods such as ball milling, laser ablation, pyrolysis, photolithography, electron beam lithography, and acid etching.^[3d]^In contrast, the bottom‐up approach (Figure 2C) involves assembling noble metal atoms, molecules, or clusters into nanomaterials with specific structures in suitable environments. This is achieved through chemical reduction, photochemical processes, electrochemical methods, and even biological approaches, offering a simple way to create nano‐bio‐interfaces.^[5a]^

#### 2.3.1 Top‐Down Fabrication

Top‐down methods have been developed for the synthesis of NMNs, including ball milling, laser ablation, and pyrolysis, among others.^[3d]^ These methods have a significant impact on the size, shape, and physical properties of the resulting nanoparticles, depending on factors such as grinding type, grinding medium, atmosphere, strength, temperature, and time.[^3^, ^5^] For example, Debnath et al. reported a solid‐state, high‐speed vibration milling approach using sodium borohydride as the reducing agent and PVP as the protecting agent to fabricate Au and Ag nanoparticles at room temperature.^[^ ^39^ ^]^ Laser ablation involves the use of high‐energy lasers to melt and evaporate material, depositing metallic nanoparticles on substrates or in solutions. The parameters of the laser, such as pulse duration, wavelength/energy density, and the physical properties of the material, play a crucial role in this process.^[^ ^40^ ^]^ Pyrolysis is another important method in which noble metal precursors are impregnated or deposited onto a suitable carrier and then burned at high temperatures to decompose organometallic oxides.^[5a]^ Additionally, there are other top‐down techniques such as photolithography, electron beam lithography, vapor deposition, and acid etching.^[3d]^ Overall, top‐down nanofabrication techniques offer advantages in terms of high fidelity and controllability. However, they may result in imperfect surface structures of the metal nanoparticles, which can affect their physical and chemical properties. Moreover, the high pressure and temperature requirements of these methods make them relatively expensive and energy‐intensive.

#### 2.3.2 Bottom‐Up Synthesis

In the bottom‐up process of synthesizing nanoparticles, metal ions are reduced from an ionic state to an atomic state by a reducing agent. These atoms then come together to form small clusters, which collide with each other and grow into larger clusters. Once a cluster reaches a stable state, a crystal nucleus is formed, and continuous growth leads to the formation of noble metal nanoparticles. In solution, these nanoparticles can further coalesce and aggregate into larger particles, lowering the energy level of the system. To prevent aggregation and maintain the dispersion of colloidal nanoparticles, protective agents, such as charge‐inducing or steric hindrance‐forming agents, are used to create a coating on their surface.

Typical bottom‐up methods include chemical reduction, photochemical, and electrochemical methods. In chemical reduction methods, different types of noble metal nanoparticles are obtained by using metal precursors, reducing agents, and capping agents (e.g., PVA, PEG, and PVP) that facilitate electron transfer.[^3^, ^41^] The choice of appropriate capping agents helps control the size, morphology, and stability of the nanoparticles, limiting their growth and preventing aggregation. Photochemical methods utilize optically active precursors to generate high‐energy intermediate states under light radiation, leading to the controlled formation of nanoparticles in the presence of a protective agent. The underlying photochemical process involves three stages including induction, growth, and maturity, which are carried out under milder and cleaner conditions compared to chemical reduction. The morphology and functionality of nanomaterials can be regulated by adjusting the experimental parameters in the photochemical synthesis.^[^ ^42^ ^]^ Electrochemical methods employ electrochemical techniques to apply a fixed potential or current in a liquid medium, resulting in various processes such as electrodeposition, cathodic etching, electrochemical redox, and electrochemical dealloying for the synthesis of nanoparticles. Electrochemical preparation and analysis methods are relatively inexpensive and versatile compared to chemical or physical methods. The electrochemical methods are particularly suitable for creating noble metal thin films, while real‐time control of the electrochemical process can be achieved through monitoring the current or voltage of the electrochemical workstation.^[^ ^43^ ^]^

Apart from ligand‐assisted growth, other bottom‐up methods, such as biological‐templated synthesis, seeded growth, and crystal phase transformation, have been developed. These methods allow the design of building blocks that facilitate the assembly of nanoparticles with specific functions. However, it should be noted that synthetic conditions involving high temperatures, non‐natural protectants, or intense agitation can potentially pose environmental concerns and require significant energy consumption.

## 3 Functionalized Noble Metal Nanomaterials with Biomolecules

NMNs can interact with biomolecules through DNA‐templated construction and peptide‐templated construction. These interactions between NMNs and biomolecules provide a basis for creating functional interfaces at the nanoscale, which hold significant potential for various biomedical and biotechnological applications.

### 3.1 DNA‐Templated Construction

DNA nanotechnology has revolutionized bottom‐up nanofabrication by leveraging the unique properties of DNA. DNA serves not only as a carrier of genetic information but also as a versatile tool for nanostructural engineering and self‐assembly. Through modification of DNA length, sequence, and intra‐ or intermolecular hybridization events, the size and shape of DNA can be adjusted to meet specific requirements.^[^ ^44^ ^]^ The functional groups on DNA backbones make them ideal biological templates for controlling the nucleation and growth of nanocolloids by binding metals or metal ions to specific sites. Moreover, the base‐pairing specificity of DNA enables precise arrangement of nanoparticles through self‐assembly, resulting in well‐defined DNA nanostructures. These DNA templates greatly enhance scalability, programmability, and functionality in the construction of noble metal nanostructures.

There are two main approaches to DNA metallization. In the first approach, metal cations initially bind to the phosphate backbone of DNA through coordination or electrostatic interactions. These bound metal ions are then chemically or photochemically reduced to form small clusters, which subsequently grow into nanoparticles. To prevent aggregation, the nanoparticles are covered with negatively charged DNA strands. The second approach involves using pre‐synthesized DNA nanostructures with specific sizes and structures as templates for shape‐controlled metallized objects.^[3c]^ DNA origami technology, for example, allows the folding of long single‐stranded DNA scaffolds into arbitrary shapes using spiked oligonucleotides, enabling the fabrication of complex nanostructures.

**Figure 3:**
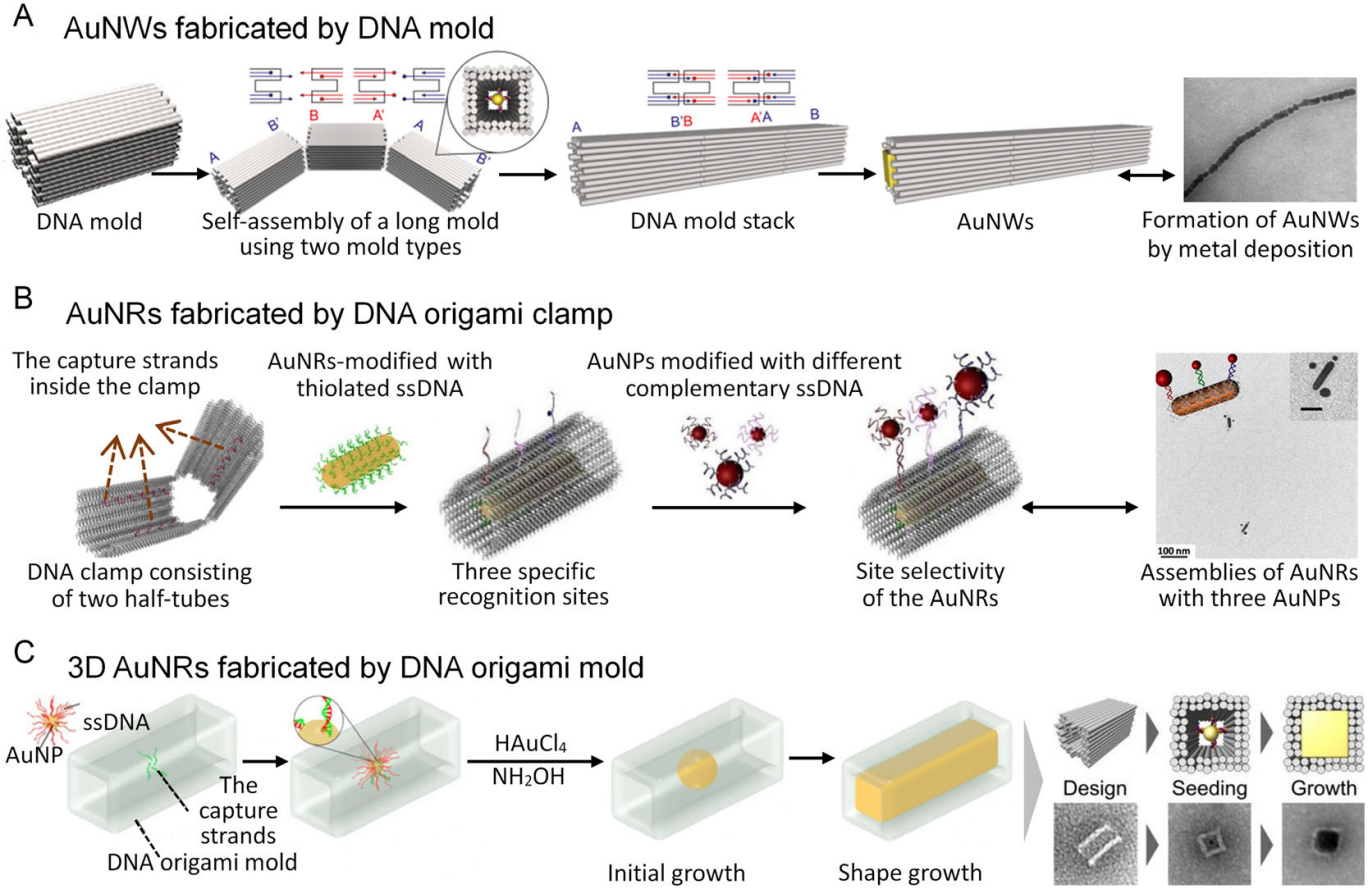
Construction of NMNs with DNA template. (A) Schematic fabrication and TEM image of gold nanowires (AuNWs) with a DNA origami mold. Reproduced with permission.^[^ ^46^ ^]^ Copyright 2018, American Chemical Society. (B) Schematic surface functionalization and TEM image of Au nanorods (AuNRs) with programmable DNA valences on the outer DNA origami clamp surface. Reproduced with permission.^[^ ^47^ ^]^ Copyright 2016, American Chemical Society. (C) Schematic assembly and TEM images of user‐specified 3D shapes of AuNPs via casting growth with a DNA origami mold. Reproduced with permission.^[^ ^48^ ^]^ Copyright 2014, American Chemical Society.

In recent years, various single and hybrid NMNs have been successfully fabricated using DNA nanotechnology.^[^ ^45^ ^]^ For instance, highly homogeneous and conductive gold nanowires have been created by using linear DNA mold superstructures obtained through self‐assembly (**Figure** **3**A).^[^ ^46^ ^]^ By employing a DNA origami clamp, gold nanorods can be modified with specific surface recognition sites,^[^ ^47^ ^]^ leading to the construction of well‐defined heterostructures with controlled chemical valence (Figure 3B). A general strategy known as DNA casting has been developed to design and synthesize inorganic nanostructures with arbitrarily prescribed 3D shapes (Figure 3C),^[^ ^48^ ^]^ including gold and silver nanoparticles with different cross‐sectional geometries, enabling potential applications in biosensors, photonics, and nanoelectronics. Additionally, 3D DNA origami structures have been utilized as templates for shaping gold clusters into desired structures such as nanocuboids, nanodonuts,^[^ ^49^ ^]^ and polymerized nanorods, providing a straightforward approach for generating complex structures with high precision.

### 3.2 Peptide‐Templated Construction

In addition to the diverse range of natural and artificial peptide sequences, peptide templates offer unique opportunities for the controlled formation of nanoparticles due to their functional and structural modularity, molecular recognition, and self‐assembly properties. Similar to DNA templates, peptide templates possess specific active groups on their molecular surfaces, such as amino, sulfhydryl, and hydroxyl groups, which allow them to interact with metal ions. Through different adsorption motifs and interactions between peptides and specific metal surfaces, peptide templates can precisely control the size, shape, structure, and surface properties of nanoparticles through bottom‐up approaches. The synthesis of bioinorganic composite nanomaterials using biological templates generally involves three steps: 1) selecting a specific spatial structure of biological molecules as templates; 2) modifying the template molecules through physical and chemical methods to combine with metal precursors and form a specific structure; and 3) reducing the metal ions on the template surface with a reducing agent to obtain nanomaterials with great potential in biomedical applications. Furthermore, the high electronic conductivity of peptide templates can enhance the catalytic activity of noble metal catalysts in photocatalysis and electrocatalysis.^[^ ^50^ ^]^

**Figure 4:**
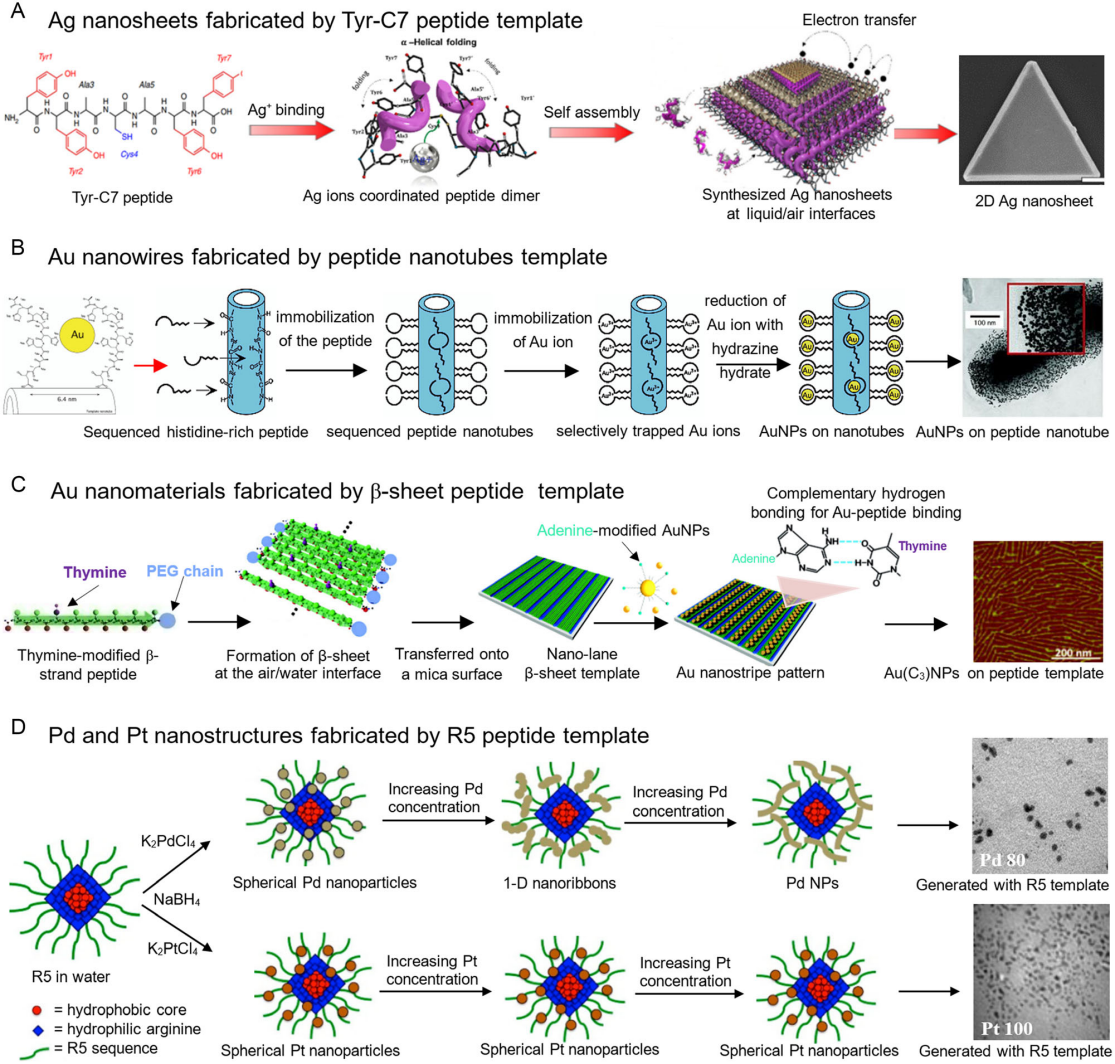
Construction of NMNs with polypeptide template. (A) Schematic growth process and scanning electron microscopy (SEM) image of layer‐by‐layer Ag nanosheets using the Tyr‐C7 peptide template. Reproduced with permission.^[^ ^51^ ^]^ Copyright 2020, American Chemical Society. (B) Schematic growth process and TEM image of Au nanowires fabricated by the peptide nanotube template. Reproduced with permission.^[^ ^52^ ^]^ Copyright 2003, American Chemical Society. (C) Schematic assembly process and Atomic force microscopy (AFM) image showing a unique 2D assembly pattern of Au nanomaterials on a *β*‐sheet monolayer template through nucleobase pairing. Reproduced with permission.^[^ ^53^ ^]^ Copyright 2011, American Chemical Society. (D) Schematic generation and TEM images of Pd and Pt nanostructures employing the R5‐based template. Reproduced with permission.^[^ ^54^ ^]^ Copyright 2013, American Chemical Society.

Significant progress has been made in the preparation of gold, silver, palladium, and platinum nanostructures with different morphologies using peptide templates. For example, Lee et al. successfully controlled the crystal growth and morphology of Ag nanomaterials using a tyrosine‐rich α‐helical 7‐mer peptide template (**Figure** **4**A).^[^ ^51^ ^]^ They embedded Ag ions layer‐by‐layer in the assembled structure of the peptide, resulting in high‐quality single‐crystalline Ag nanosheets at room temperature. Efficient electron transfer between the independent metal nanosheets was induced by the peptide template, improving the electrochemical properties. Djalali et al. fabricated Au nanowires using sequenced peptide nanotubes as templates (Figure 4B).^[^ ^52^ ^]^ Histidine‐rich peptide molecules were assembled to selectively trap Au ions and initiate nucleation, resulting in the growth of Au nanocrystals inside and on the outer surfaces of the nanotubes. The size and packing density of the Au nanocrystals were controlled by the conformations and charge distributions of the histidine‐rich peptides, which were regulated by pH and Au ion concentration in the growth solution. Nonoyama et al. arranged gold nanoparticles (AuNPs) on an amphiphilic *β*‐sheet peptide template (Figure 4C).^[^ ^53^ ^]^ By introducing thymine onto the hydrophobic surface of the peptide sequence, a *β*‐sheet monolayer was formed at the air/water interface and transferred to a mica surface. Adenines were chemically modified onto the AuNPs, allowing for spontaneous nucleobase pairing with thymine on the *β*‐sheet template. This unique 2D nucleobase pattern on the template controlled the assembly structure of the AuNPs. Bhandari et al. synthesized Pd nanostructures with varying morphologies using a peptide template (Figure 4D).^[^ ^54^ ^]^ The morphological changes depended on the ratio of Pd/peptide, resulting in a stepwise transformation from nanospheres to highly branched nanoparticles in networks. In contrast, no structural changes were observed for spherical Pt nanoparticles regardless of the ratio of Pt/peptide. These differences in structural morphology were attributed to various interactions among the metal, peptide, and peptide‐peptide interactions, as well as the rates of nanostructure nucleation, growth, and aggregation.

## 4 Noble Metal Nanomaterials Meet with Biological Systems

In the past two decades, extensive attention has been paid to the absorption, distribution, metabolism, and removal of NMNs in biological systems, as well as their impact on the physiological functions of target organs. This is due to their potential applications in various fields including therapeutics, diagnostics, and bioimaging. NMNs are surrounded by biomolecules in biological fluids and reach saturation at nano‐bio‐interfaces through interactions with molecules such as phospholipids, proteins, and DNA. These nano‐bio‐interactions occur during biological modification of NMNs and can influence their binding events with cells, ultimately changing their properties. Once inside cells, NMNs show a tendency to localize in specific organelles, affecting particular cell regions.^[^ ^55^ ^]^ It is critical to study the physical and chemical interactions between NMNs and biological systems in detail at the biofluidic, biomolecular, cellular, and tissue/organism interfaces. Such studies have attracted great attention due to the potential health‐related applications of NMNs, which include drug delivery, disease detection, bio‐diagnosis, biomedicine, and therapy. Overall, understanding these interactions is essential for optimizing the design of NMNs for specific applications (**Table** **1**), and for ensuring their safety and efficacy in biomedical contexts.

**Table 1:** Biomedical applications of NMNs

NMNs	Applications	Advantages	References
AuNPs	Antibacterial	Acid‐responsive PTT systema)	[170]
AuNPs	Antibacterial and bioimaging	Multiple routes of administration and fluorescent tracer	[171]
AuNPs	Antibacterial	Oral administration and in vivo synthesis	[172]
AuNPs	Antibacterial	Deoxyribonuclease functionalized AuNCs and effectively remove biofilms	[173]
Aminobenzeneboronic acid modified AuNCs	Antibacterial and self‐monitor	Self‐monitoring residual dosage	[174]
PolySC_4_AP/FGGC@AuNC assemblies^b)^	Mitochondrial imaging	Host‐guest assembly and high quantum yield	[175]
Pt(IV)‐ALA‐Chitosan‐AuNCs^c)^	Anticancer and bioimaging	Traceable nanocluster and combinatorial therapy	[176]
AuNCs/QGDs/MPA/PEI^d)^	Anticancer	pH‐ and near‐infrared‐responsive and combinatorial therapy	[177]
AgNCs/AgNPs	Antibacterial and bioimaging	Versatile antimicrobial	[178]
DNA‐templated AgNCs	Anticancer and bioimaging	Combinatorial therapy and fluorescence tracing and chemo drug delivery capabilities	[179]
Peptide‐protected AgNCs	Antimicrobial	Green synthesis	[180]
PtNCs	Fluorescent probe	Detecting and imaging HER2^e)^	[181]
Pillararene‐functionalized RhNPs	Antibacterial	Photothermal sterilization and efficient catalytic ability (reduce nitrophenols and azo dyes)	[182]
uRh‐mSNO NPs^f)^	Anticancer	Autophagy inhibition effect and peroxidase mimic and lysosome dysfunction	[183]
PdNPs/covalent organic framework^g)^	Biosensor and bioimaging	Multicolor imaging	[184]
Hyaluronate‐modified Au@PtNPs	Anticancer and bioimaging	Transdermal delivery and photoacoustic imaging	[169]
Yeast microcapsule‐derived Au/PtNPs	Cancer immunotherapy	Combinatorial therapy and chemodynamic/photothermal synergistic treatment	[185]
Core–shell structured Au@Pd	Anticancer	ROS augmentation‐induced mild PTT	[186]

a) PTT: photothermal therapy; ^b)^PolySC4AP/FGGC: polysulfonatocalix[4]areneAP/phenylalanine‐glycine‐glycine‐cysteine; ^c)^ALA: aminolevulinic acid; ^d)^GQDs/MPA/PEI: graphene quantum dots/mercaptopropionic acid/polyethyleneimine; ^e)^HER2: human epidermal growth factor receptor 2; ^f)^uRh‐mSNO NPs: ultra‐small rhodium‐methoxypolyethylene glycol‐S‐nitrosothiols nanoparticles; and ^g)^PdNPs/covalent organic framework: palladium nanoparticles/carboxymethyl cellulose‐modified covalent organic framework hydrogel.

### 4.1 Building Strategies for NMNs at Nano‐bio‐interface

Nano‐bio‐interface is achieved by conjugating NMNs, such as Au, Pt, and Ag, to biomolecules, enabling a wide range of applications in bioimaging, biosensing, targeted drug delivery, enzyme immobilization, and catalysis.^[^ ^56^ ^]^ Several strategies have been developed to attach NMNs to biomolecules, including electrostatic adsorption, covalent grafting, and electrochemical bioconjugation. Inorganic‐organic nano‐bio‐interface structures typically exhibit a core–shell configuration, with a noble metal core surrounded by a covalently attached monolayer of biomolecules. For example, thiolated AuNPs can interact strongly with thiol‐modified DNA or cysteine‐containing peptides/proteins to form AuNP‐biomolecule conjugations via covalent bonding. Electrostatic interactions can also be used to adsorb proteins onto negatively charged AuNPs.

#### 4.1.1 Electrostatic Adsorption

Binding biomolecules to noble metal nanoparticles through electrostatic adsorption is a simple and easy method that has gained extensive interest in recent years. This method relies on the opposite charges on the surfaces of biomolecules and NMNs to allow them to combine easily, enabling the construction of nano‐bio‐interfaces with a range of biomolecules, including proteins, enzymes, and DNA. For example, Shenton et al. reported the electrostatic adsorption of negatively charged AuNPs or AgNPs with positively charged immunoglobulin G using pH adjustment below the iso‐electric point at pH≈7.^[^ ^57^ ^]^ Similarly, Rospendowski et al. reported the electrostatic adsorption of cytochrome P‐450 enzymes with citrate‐reduced AgNPs.^[^ ^58^ ^]^

**Figure 5:**
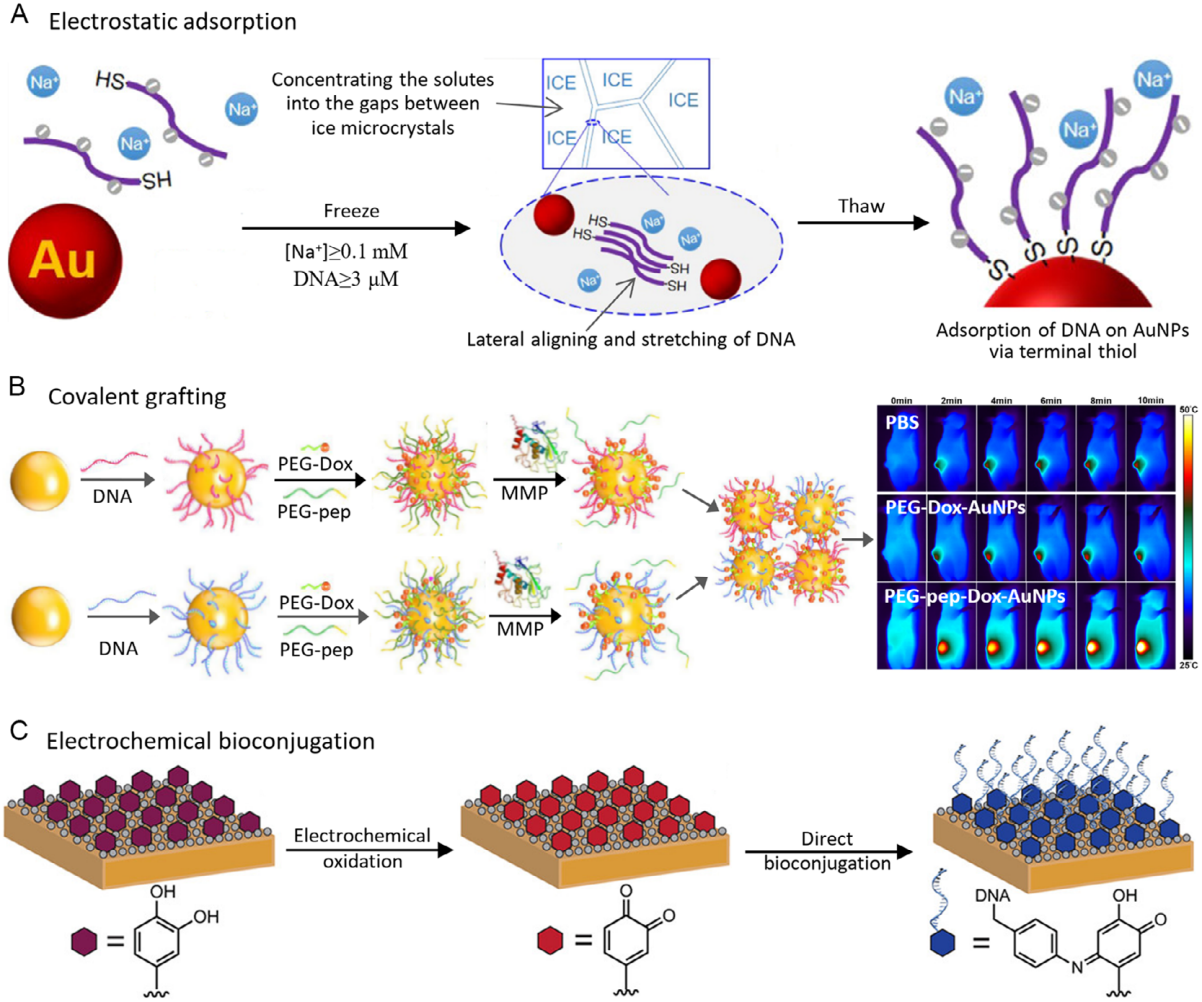
Building strategies for NMNs at nano‐bio‐interface. (A) Schematic illustration of freeze‐directed conjugation of thiolated DNA on citrate‐capped AuNPs as a building strategy. Reproduced with permission.^[^ ^60^ ^]^ Copyright 2019, American Chemical Society. (B) Schematic illustration of a nanoplatform utilizing matrix metalloproteinase (MMP)‐responsive AuNPs for tumor‐targeted photoacoustic imaging‐guided photothermal therapy. Reproduced with permission.^[^ ^65^ ^]^ Copyright 2019, Elsevier. (C) Schematic illustration of direct electrochemical bioconjugation reaction to couple nucleic acids to the gold electrode surface. Reproduced with permission. Copyright 2017, American Chemical Society.^[^ ^66^ ^]^

DNA molecules with a negatively charged phosphoric acid skeleton can be electrostatically adsorbed onto positively charged noble metal nanoparticles to form a DNA/NMNs nano‐bio‐interface. Addition of salt can also help screen charge repulsion when both DNA and NMNs are negatively charged, allowing for successful attachment. Various approaches have been reported to securely attach DNA molecules to surface‐functionalized AuNPs. For example, Nakao et al. achieved a DNA/AuNPs nanostructure with a long‐range order on substrate by strongly attaching DNA to the aniline‐capped AuNPs with positive charge and aromatic ring via electrostatic and *π–π* interactions.^[^ ^59^ ^]^ Liu et al. studied the thiolated DNA attachment on citrate‐capped AuNPs under freezing conditions (**Figure** **5**A),^[^ ^60^ ^]^ achieving a high DNA density at high DNA concentration upon freezing through stretch and aligning DNA confirmation to avoid cross‐linking of two AuNPs by the same DNA sequence. Zhang et al. proposed a model to rationalize the adsorption/desorption behavior of DNA onto citrate‐capped AuNPs based on electrostatic repulsion and studied the adsorption kinetics, capacity, and isotherms of DNA,^[^ ^61^ ^]^ demonstrating that adding salt could increase both the adsorption and desorption rates of DNA from AuNPs.

#### 4.1.2 Covalent Grafting

Biomolecules can be adsorbed onto the surface of NMNs through electrostatic interaction, but weak electrostatic adsorption force may cause them to fall off easily. To address this issue, chemical coupling of biomolecules to the surface of NMNs via covalent bonding can be used. For example, Zhao et al. used rolling circle amplification to graft long single‐stranded DNA molecules with repetitive sequence units onto AuNPs, forming periodic 3D noble metal nanostructures.^[^ ^62^ ^]^ Higuchi et al. prepared peptide‐coated AuNPs that form a fibril assembly due to dipole–dipole interaction between surface peptides,^[^ ^63^ ^]^ and Quan et al. grafted the trans‐acting activator of transcription peptide onto AuNPs for translocation across the cell membrane and drug delivery.^[^ ^64^ ^]^

Similarly, Yang et al. developed matrix metalloproteinase‐responsive AuNPs for tumor‐targeted photoacoustic imaging‐guided photothermal therapy and drug delivery.^[^ ^65^ ^]^ The nanoplatform was coated with PEG via a matrix metalloproteinase‐cleavable peptide and tethered with doxorubicin via a thermal‐labile linker (Figure 5B). The DNA hybridization‐induced assembly of the nanoprobes exhibited prolonged tumor retention and strong near‐infrared absorption for deep‐tissue imaging and therapy. The doxorubicin was released rapidly in response to localized temperature elevation for synergistic chemo‐photothermal therapy. This nanoplatform demonstrated significantly enhanced efficiency in photoacoustic imaging and photothermal conversion upon near‐infrared irradiation.

#### 4.1.3 Electrochemical Bioconjugation

Electrochemical approaches offer a promising method to create nano‐bio‐interfaces because of their simplicity, rapid operation, adjustable reaction time, and tunable binding rate between biomolecules and NMNs. Recently, several noteworthy research findings have emerged in this field. For instance, Furst et al. introduced an electrochemically activated bioconjugation reaction to couple nucleic acids to gold electrode surfaces quickly by oxidizing aniline‐modified DNA to o‐quinones at a specific applied potential (Figure 5C).^[^ ^66^ ^]^ This approach facilitated the formation of DNA monolayers within minutes without requiring additional reagents. Additionally, Rant et al. developed an innovative approach that allowed for dynamic control of the orientation of short oligonucleotide strands tethered to gold electrodes in an electrolyte solution.^[^ ^67^ ^]^ Drummond et al. described various methods to indirectly oxidize target DNA using electrochemical mediators,^[^ ^68^ ^]^ which successfully detected trinucleotide repeats and gene overexpression in tumors. These electrochemical approaches have advanced the field of nanobiotechnology through their versatility and powerful tools for constructing nano‐bio‐interfaces, investigating molecular dynamics, enabling label‐free biosensing, and detecting specific DNA sequences.

### 4.2 Configuration of NMNs at Nano‐bio‐interface

Studying the physical and chemical interactions of NMNs at different biological interfaces is essential for understanding their behavior and optimizing their applications. At the level of biofluidics, investigations focus on how NMNs interact with biological fluids, how they are transported within the body, and how they are cleared from circulation. At the biomolecular level, researchers examine the binding affinity and mechanisms between NMNs and biomolecules, which can influence the stability and functionality of both the nanoparticles and the biomolecules. Cellular studies shed light on the internalization pathways, subcellular localization, and potential effects of NMNs on cellular processes and functions. Finally, investigations at the tissue/organism level aim to elucidate how NMNs interact with specific tissues or organs, and how these interactions may contribute to therapeutic outcomes or diagnostic capabilities.

#### 4.2.1 Biofluidic Interface

**Figure 6:**
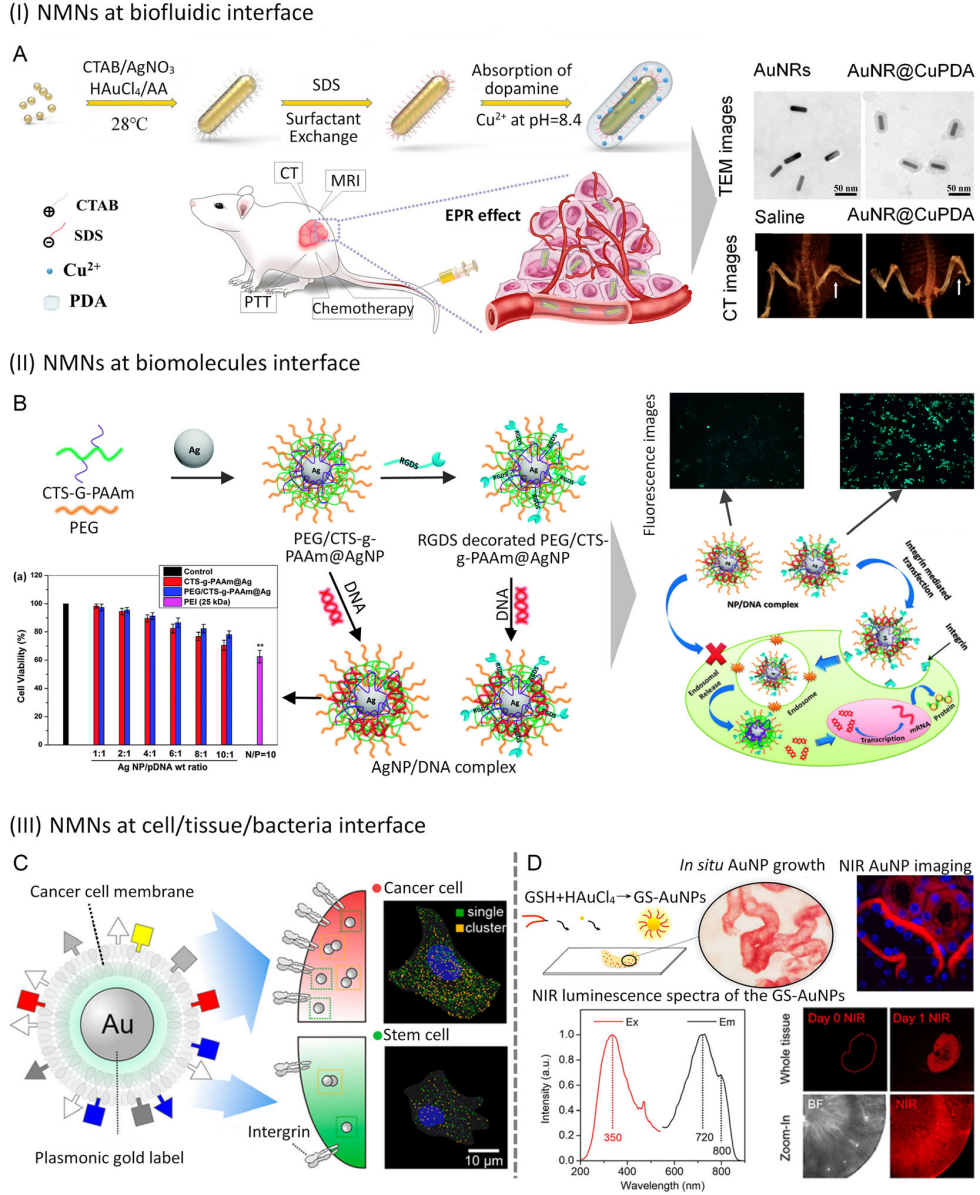
Configurations of NMNs at nano‐bio‐interface. (I) NMNs at the biofluidic interface: (A) Metabolic pathways of AuNPs in vivo. Reproduced with permission.^[^ ^72^ ^]^ Copyright 2017, American Chemical Society. (II) NMNs at the biomolecular interface: (B) Preparation of biofunctionalized silver nanoparticles (AgNPs). Reproduced with permission.^[^ ^76^ ^]^ Copyright 2015, The Royal Society of Chemistry. (III) NMNs at the cell/tissue/organism interface: (C) Plasmonic imaging performance of interactions between cancer cell membrane‐coated AuNPs and living cells at the single‐cell level, and (D) in situ growth of AuNPs in kidney tissues. Reproduced with permission.^[^ ^82^ ^]^ Copyright 2020, American Chemical Society. Reproduced with permission.^[^ ^83^ ^]^ Copyright 2019, American Chemical Society.

Once NMNs are introduced to biological fluids like blood and interstitial fluid, they immediately adsorb proteins to their surface, forming a protein corona that gives NMNs biological properties.^[^ ^69^ ^]^ This results in an initial nano‐bio‐interface that changes dynamically as NMNs move onto or into cells.^[^ ^70^ ^]^ However, limitations such as inflammatory responses, cytotoxicity, unexpected distribution and clearance from the body, and insufficient delivery to specific targets hinder their application in vivo.^[^ ^71^ ^]^ To address this, dopamine‐coated AuNPs (MSA AuNPs@DPA aggregates) were developed for investigating their metabolic pathways in vivo. These aggregates had a long circulation time, low toxicity, and excellent CT (computed tomography) absorption value, making them a potential prospect for in vivo applications (**Figure** **6**A).^[^ ^72^ ^]^

Monitoring fluid flow in living organisms is crucial for understanding dynamic processes. Velocity measurement in biological fluids is essential for detecting and treating circulatory diseases related to abnormal flow. Ahn et al. modified the surface of ≈20 nm AuNPs to label the lignin vessels as a flow‐tracking sensor in non‐transparent biological fluids for monitoring the flow phenomenon of the perforated end wall of xylem blood vessels in rice.^[^ ^73^ ^]^ This technique quantifies the transport of sap in the lignin vessel and helps to solve the mysteries surrounding plant metabolism.

#### 4.2.2 Biomolecular Interface

Proteins, as a significant class of biological macromolecules, are composed of amino acid residues with varying size, shape, and net charge. The hydrophobicity of a protein surface is determined by the exposed amino acid residues, which can influence their interactions with NMNs. The conjugation of NMNs with proteins has been employed in biosensing and early disease detection. For instance, Ge et al. utilized strong Au‐S binding to assemble two‐photon dye‐labeled peptide sequences onto AuNPs’ surface, creating a fluorescence resonance energy transfer strategy‐based two‐photon excited nanosensor.^[^ ^74^ ^]^ This nanosensor enabled the detection of caspase‐3 in live cells and tissues. Similarly, Kosaka et al. combined mechanical and optoplasmonic transduction to detect extremely low concentrations of cancer biomarkers like cancer embryonic antigen (CEA) and prostate‐specific antigen (PSA) in blood.^[^ ^75^ ^]^ They achieved this by functionalizing a silica cantilever with capture antibodies, followed by immunoreaction with detection antibodies tethered to 100 nm AuNPs. The method allowed for mass measurement of captured AuNPs by the silicon cantilever and enhanced the plasmonic signal from the AuNPs when serving as both a mechanical resonator and an optical cavity. Utilizing this approach, the detection limits for CEA and PSA biomarkers in serum reached an impressive 1 × 10^−16^ g mL^−1^, at least seven orders of magnitude lower than routine clinical practice.

DNA molecules, whether single‐ or double‐stranded, can interact with NMNs through electrostatic, *π*–*π* stacking, and hydrophobic interactions, which depend on the properties of both the DNA and NMNs. These interactions have biomedical applications such as gene delivery and DNA detection. Sarkar et al. developed PEG‐stabilized chitosan‐g‐polyacrylamide (CTS‐G‐PAAm)‐modified AgNPs (Figure 6B),^[^ ^76^ ^]^ which exhibited enhanced DNA complexation capability with increasing AgNPs/pDNA weight ratio. Furthermore, when Arg‐Gly‐Asp‐Ser peptide molecules were immobilized on PEG/CTS‐G‐PAAm@AgNPs, these biofunctionalized AgNPs served as efficient nonviral carriers in gene therapy and significantly improved gene transfection efficiency in HeLa and A549 cells.

#### 4.2.3 Cell/Tissue/Organism Interface

NMNs have the ability to interact with cells at the cellular level, making it important to investigate and understand the interaction between the nanoparticles and cells.^[^ ^77^ ^]^ The nanoparticle‐cell interactions are influenced by the size, shape, surface chemistry, and surface charge distribution of NMNs.^[^ ^70^ ^]^ In particular, NMNs can interact directly with cell membranes, specifically with phospholipids which make up the membrane. The interaction depends on the surface hydrophobicity of the NMN, and can occur with either the negatively charged heads or the hydrophobic tails of the membrane.^[^ ^55^ ^]^ Elfiky et al. conducted molecular dynamics simulations to investigate the process of citrate‐modified AuNPs interacting with cancer cell membranes. The simulations revealed that when the AuNPs approached the cell membrane, the interaction between the two resulted in the rupture of the phospholipid membrane, forming pores. Subsequently, the AuNPs entered the cells through these pores. However, the specific interaction mechanism leading to the formation of these pores on the phospholipid cell membrane was not accurately explained by the simulation data.^[^ ^78^ ^]^ Nonetheless, the presence of these pore structures is one of the reasons why the therapeutic effects of drugs are enhanced in the presence of noble metal nanomaterials.^[^ ^79^ ^]^ Moreover, the interaction between NMNs and cell membranes is closely associated with the nanomaterials’ morphology. Lins et al. performed simulations to investigate the interaction between gold nanorods with different aspect ratios and cell membranes.^[^ ^80^ ^]^ The simulation results demonstrated that noble gold nanomaterials influenced the expansion isotherm of the cell membrane, indicating phase separation at the interface of lipids and nanomaterials. The occurrence of this phenomenon was found to be dependent on the size of the nanomaterials. Utilizing the interaction between nanomaterials and cell membranes, researchers have developed a system of cell membrane‐coated nanoparticles, capitalizing on this unique property. Cell‐membrane‐camouflaged nanoparticles (CMC‐NPs) combine the physiochemical characteristics of nanoparticulate cores with biological features of membrane‐providing cells. For example, red blood cell membrane coating can help extend the circulation time of nanoparticles in vivo by evading immune clearance, while platelet membrane‐encapsulated nanoparticles can effectively target damaged vascular systems through selective binding.^[^ ^81^ ^]^ CMC‐NPs have been fabricated using different membrane sources such as blood cells, stem cells, pancreatic beta cells, and cancer cells.

Recent studies have focused on the nano‐bio‐interface of NMNs. Xie et al. constructed plasmonic CMC‐NPs by coating AuNPs with cancer cell membranes, and studied the interactions between CMC‐NPs and living cancer or stem cells at a single‐cell level (Figure 6C),^[^ ^82^ ^]^ characterizing the interface between the nanoparticles and cells, and providing new ideas for future research. Peng et al. reported the in situ ligand‐directed growth of glutathione‐protected AuNPs (GS‐AuNPs) in biological tissues (Figure 6D),^[^ ^83^ ^]^ demonstrating the potential of AuNPs in biological imaging. Verma et al. developed a chemical nose biosensor based on AuNPs to detect,^[^ ^84^ ^]^ identify, and quantify species of pathogenic bacteria. The chemical nose biosensor was able to detect and identify different species of bacteria with high accuracy, making it a promising tool for rapid diagnosis of infectious diseases.

## 5 Interfacial Interactions of NMNs with Biological Systems

The interactions between NMNs and biomolecules are influenced by various factors, including the nanostructured properties of NMNs (such as size, shape, surface charge, and surface coating) and the compositions and molecular structures of biomaterials (such as proteins with primary and advanced conformations). Extensive research has been conducted on the thermodynamic and kinetic adsorption of biomaterials (e.g., DNA, proteins, and peptides) or organisms (e.g., cells, bacteria, and tissues) onto NMNs surfaces. The interaction at the NMNs‐biological interface is driven by multiple forces, including hydrogen bonding, metal coordination, stereo‐selection, hydrophobic interactions, and electrostatic interactions. In recent years, there has been increased focus on studying chemical reactions and antibacterial properties at the nano‐bio‐interface. These investigations aim to deepen our understanding of the diverse interactions and potential applications of NMNs in biological environments.

### 5.1 Thermodynamic and Kinetic Adsorption of NMNs to Biomaterials or Organisms

The investigation of interfacial interactions between NMNs and various biological components is crucial for understanding and controlling the role of NMNs in biological systems. To achieve this, extensive basic research is required to develop interaction models that consider the thermodynamic and kinetic adsorption of biomaterials onto NMNs surfaces. These models should not only incorporate the effects and properties of NMNs and biomaterials but also consider interaction mechanisms such as binding affinity (affinity constant between biomaterials and NMNs), binding cooperativity, and binding thermodynamics (including changes in free energy, enthalpy, entropy, and heat capacity). By focusing on these aspects, we can gain valuable insights that will enable precise control of NMN behavior in biological systems.

#### 5.1.1 Adsorption of Biomolecules to NMNs

The size and morphology of NMNs, as well as the composition and structure of biomaterials, play a significant role in determining the adsorption rate, strength, and site between them.^[^ ^85^ ^]^ Shao et al. demonstrated that the selective adsorption of glycine onto AuNPs of varying sizes was attributed to the different facet areas and surrounding media.^[^ ^86^ ^]^ The types of nucleotide bases and the length of nucleotides also influence the adsorption interactions with NMNs.^[^ ^87^ ^]^ Environmental factors have been shown to affect adsorption as well. For example, Mahmoudi et al. observed that Au nanorods altered the protein corona composition under both plasmonic heating and conventional heating conditions.^[^ ^88^ ^]^ Conducting further basic research is necessary to identify commonalities and understand the complex and diverse factors involved. This knowledge will facilitate the design of materials with specific structures to achieve desired functions.

#### 5.1.2 Interfacial Interaction Mechanisms

The adsorption of biomolecules onto NMNs has been extensively studied in terms of binding affinity, binding cooperativity, and thermodynamics. Binding affinity refers to the strength of interaction between target biomolecules and NMNs. Upon injection into the bloodstream, nanomaterials rapidly bind to highly abundant proteins (such as human serum albumin, fibrinogen, apolipoprotein A1, and immunoglobulin) present in the serum. Over time, these proteins are gradually replaced by more strongly adsorbed proteins, such as human alpha‐1‐acid glycoprotein.^[^ ^89^ ^]^ Determining the affinity constants between NMNs and biomolecules is crucial for predicting the adsorption process in biological systems. This information provides valuable insights into the interactions between NMNs and biomolecules, offering a foundation for understanding their behavior in biological environments.

#### 5.1.3 Effect of Binding Energy

The interaction between gold nanoparticles (AuNPs) and the blood compartment is influenced by the binding energy of their surface ligands. AuNPs stabilized with different ligands, such as citrate, polyallylamine, cysteamine, dihydrolipoic acid, and polyethylene glycol were synthesized and characterized. The formation of complexes between AuNPs and model proteins, namely human albumin and human fibrinogen, exhibited various behaviors, ranging from corona formation to aggregation. By studying protein fluorescence quenching at different temperatures and AuNP concentrations, we determined the thermodynamic parameters were determined to describe these interactions. Additionally, the hemolytic properties of AuNPs were explored, observing that the induction of hemolysis varied depending on the formation of protein coronas. These findings provide insights into the ideal surface ligand for blood‐compatible AuNPs, which should be strongly bound to the gold core through thiol groups and impart a negative charge to the particles.

Binding cooperativity (*n*) describes how the presence of previously adsorbed biomolecules affects subsequent adsorption on the surface of NMNs. Specifically, when examining protein adsorption, there are three possible scenarios for the number of protein layers adsorbed onto NMNs: If *n* < 1, the association energy per particle decreases as more proteins adhere to the surface. This implies that the binding strength between NMNs and proteins weakens as additional proteins bind. If *n* > 1, the association energy gradually increases as more particles bind to the proteins. This indicates that the binding strength between NMNs and proteins strengthens with more proteins accumulating on the surface. If *n* = 1, the association energy of the complexes remains constant, regardless of the number of proteins adsorbed. In this case, the binding strength between NMNs and proteins is unchanged by further protein addition. Understanding the value of binding cooperativity (*n*) provides insight into the interactions between NMNs and proteins, which is essential for designing and controlling their adsorption behavior in biological systems.

The interaction between NMNs including AuNPs and AuNCs (gold nanoclusters) and proteins can be analyzed using the enthalpy change (Δ*H*) and entropy change (Δ*S*). When Δ*H* and Δ*S* are less than zero, it indicates an exothermic process with a decrease in entropy.^[^ ^90^ ^]^ This thermodynamically driven process suggests a favorable interaction between the NMNs and proteins. Conversely, when Δ*H* and Δ*S* are greater than zero, it signifies an endothermic process with an increase in entropy.^[^ ^91^ ^]^ This entropy‐driven process suggests that as the temperature increases, more proteins will adsorb onto the surface of the precious metal nanomaterials.^[^ ^92^ ^]^ In cases where Δ*H* is less than zero and Δ*S* is greater than zero, it represents a reaction driven by both thermodynamics and entropy.^[^ ^91^ ^]^ This type of interaction between noble metals and proteins often occurs spontaneously (**Table** **2**). However, when Δ*H* is greater than zero and Δ*S* is less than zero, the interaction between noble metals and proteins is limited, making it challenging to occur. By measuring Δ*H* and Δ*S* during the kinetic process of NMNs and proteins, we can determine the driving force behind their interaction at the interface between nanomaterials and proteins. This knowledge allows us to modify the surface properties of NMNs to selectively alter their interaction with proteins and other biomolecules.

**Table 2:** Preliminary prediction of the type of driving force by the sign and values of Δ*H* and Δ*S*

Nanomaterials	protein	Δ*H*	Δ*S*	Driving force
AuNPs	Chymotrypsin	−283.7	−840.0	Hydrogen bonds and Van der Waals forces^[^ ^90^ ^]^
DHLA‐AuNCs	Human serum albumin	+37.3	+215.3	Hydrophobic interactions^[^ ^91^ ^]^
DHLA‐AuNCs	Transferrin	−12.6	+47.7	Electrostatic forces^[^ ^91^ ^]^

The study of binding thermodynamics focuses on understanding the mechanism of interaction between biomolecules and NMNs. Ferreira et al. investigated the interaction between ultra‐small AuNPs and thrombin using LSPR bio‐sensing and stopped‐flow spectroscopy.^[^ ^93^ ^]^ Based on their understanding that protein–protein interactions and the interaction between AuNPs and proteins are similar to some extent, they proposed that the binding between AuNPs and thrombin initially occurs through random collisions and forms an intermediate encounter complex via electrostatic attraction. The intermediate complex then undergoes partial desolvation of the interface, removal of ions at the interface, and possible conformational changes to overcome certain energy barriers and form the final complex. Interestingly, they observed three different desorption processes during this interaction, likely caused by slight differences in size and morphology of the ultra‐small AuNPs. This illustrates the complexity of the interaction between NMNs and biomolecules and highlights the necessity of further research.

Tollefson et al. used experimental footprinting and computational molecular dynamics methods to precisely predict the orientation of peripheral membrane protein, cytochrome c, on the surface of mercaptopropionic acid‐functionalized AuNPs.^[^ ^94^ ^]^ Through MD simulations and protein footprinting, they concluded that cytochrome c can adopt two different, but specific, orientations when binding non‐rigidly to the modified AuNPs. Furthermore, the tandem use of both methods not only determined which site of the protein interacts with the NMNs, but also simulated the functional site exposed to the environment after protein binding to the NMNs, allowing for prediction of subsequent interactions of the complex to a certain extent. Tollefson et al. also used this combined method to study the interaction between cytochrome c and the cell membrane, exploring the interaction between AuNPs, peripheral membrane proteins, and the membrane. The combination of experimental and computational tools has broad utility for exploring the interaction mechanisms between NMNs and biological components, including those in complex systems.

### 5.2 Driving Forces for Interfacial Interaction

NMNs possess remarkable photothermal, electrical, and biocompatible properties, making them highly valuable in various biological applications such as imaging, drug delivery, diagnosis, and clinical treatment.^[^ ^71^ ^]^ However, the lack of understanding regarding the physicochemical properties of NMNs often leads to a range of challenges during their application, including membrane denaturation, protein denaturation, and loss of nucleic acid function. These problems are closely associated with the nano‐bio‐interface, which governs the interactions between nanomaterials and biological systems.

The interactions occurring at nano‐bio‐interfaces encompass hydrogen bond interactions, metal coordination interactions, stereo‐selective interactions, hydrophobic interactions, and electrostatic interactions. By comprehensively understanding these diverse interactions at the nano‐bio‐interfaces, we can gain better insights into the nature of these interactions. This knowledge, in turn, enables us to meticulously design nanomaterials to avoid undesired interactions with biological systems and even exploit these interactions to achieve desired biological functionalities.

#### 5.2.1 Electrostatic Interaction

**Figure 7:**
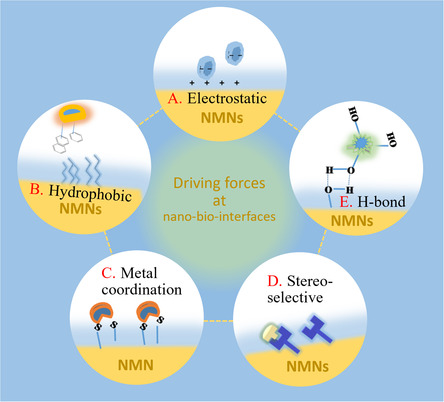
Driving forces at nano‐bio‐interfaces, including (A) electrostatic, (B) hydrophobic, (C) metal coordination, (D) stereoselective, and (E) hydrogen bond interactions.

Electrostatic interactions are common at the nano‐bio‐interface (**Figure** **7**A). Whether in vivo or in vitro, charged NMNs interact with charged biomolecules like proteins, nucleic acids, and cell membranes.^[^ ^95^ ^]^ These electrostatic interactions can both contribute to the desired functionality of NMNs and cause side effects. Upon entering the bloodstream, NMNs quickly become coated with proteins, forming protein crowns. Electrostatic interactions are primarily responsible for allowing certain biomolecules to approach NMNs from a distance. Experimental data from Wang et al. indicate that the electrostatic interaction is mainly driven by protein lysine residues,^[95a]^ and modifying these residues can alter the strength of adsorption. This presents a new strategy for designing nanobiological complexes.

It has been observed that negatively charged glycoproteins on cell membranes repel negatively charged AuNPs,^[^ ^96^ ^]^ leading to repulsive deposition. Conversely, positively charged AuNPs are attracted to the cell membrane due to electrostatic forces. However, negatively charged AuNPs continuously rebound on the cell membrane due to electrostatic repulsion until they deposit at the cell junction, disrupting cell bonds and causing leakiness in endothelial cells. Many research groups have investigated the influence of surface charge on nanomaterials at the nano‐bio‐interface and have found that it affects the number of nanomaterials on the membrane and their mode of entry. Although precise control over NMN behavior solely through surface charge modification is challenging, electrostatic interactions can be utilized to modify specific proteins or nucleic acids on the surface of NMNs, enabling targeted transport and specific functionalities.

#### 5.2.2 Hydrophobic Interaction

Hydrophobic interactions, although less common than electrostatic interactions, also play an important role in the nano‐bio‐interface as a short‐range force (Figure 7B). Since the cell membrane consists of amphiphilic phospholipid molecules, the interaction between NMNs and the cell membrane has been a focus of many research teams. Sun et al. found that negatively charged modified AuNPs face electrostatic repulsion and thus have difficulty adsorbing onto the cell membrane's surface.^[^ ^97^ ^]^ However, octanethiol‐modified AuNPs can easily adsorb to the cell membrane due to the hydrophobic interaction between the octanethiol group and the internal phospholipid bilayer. Guo et al. explored the influence of hydrophobicity on the penetration ability of AuNPs through cell membranes.^[^ ^98^ ^]^ The results showed that modifying AuNPs with hydrophobic groups increased their ability to penetrate the cell membrane. However, small‐sized AuNPs (<5 nm) can get trapped in the phospholipid bilayer due to strong hydrophobic interactions.

In addition, hydrophobicity plays other important roles. It is recognized that proteins readily adsorb onto NMN surfaces, forming a protein corona that affects the functionality of nanomaterials.^[^ ^92^ ^]^ However, Porret et al. discovered that by properly regulating the hydrophilic/hydrophobic balance of AuNPs,^[^ ^99^ ^]^ it is possible to effectively prevent biomolecule adsorption and maintain the stability of NMNs. Therefore, gaining an in‐depth understanding of hydrophobic interactions at the nano‐bio‐interface can help us modify the surface structure of nanomaterials based on interaction principles, thereby better realizing the desired functionalities of nanomaterials.

#### 5.2.3 Metal Coordination Interaction

Metal coordination refers to the interaction between metal atoms or ions and ligands through coordination bonds (Figure 7C). This phenomenon is widely observed in the nano‐bio‐interface and has significant research implications. Synthetic nano‐bio‐interfaces often utilize metal coordination to investigate the function and fate of biomolecule‐coated nanomaterials in vivo.^[^ ^100^ ^]^ For example, Pei et al. discovered that metal coordination occurs between N on DNA guanine and metal,^[^ ^101^ ^]^ which leads to the absorption of DNA on NMNs. This guanine‐dependent adsorption can be used as a basis for developing a simple DNA sequence recognition device. Studies have also found strong metal coordination between proteins and NMNs, primarily between metal and cysteine mercaptan. Proteins with less free mercaptan can be displaced by proteins with more mercaptan or even small molecules containing free mercaptan. Therefore, this provides a new method for developing stable biological complexes between proteins or other biomolecules and NMNs through metal coordination. Additionally, the presence of metal coordination increases the difficulty of studying the nano‐bio‐interface, particularly regarding the mechanism of protein crown formation.

#### 5.2.4 Stereo‐selection Interaction

Molecular conformation plays a significant role in stereoselectivity. Different conformations of molecules can result in distinct binding sites (Figure 7D). Certain conformations may encounter steric hindrance, making it difficult for them to interact with the target molecule and leading to stereoselectivity. Although there are limited reports on naked NMNs interacting with biological components through stereotactic interaction, such interactions contribute to achieving specificity and targeting of nanomaterials to some extent. This enables better functioning and reduces side effects. Zhan et al. discovered that modifying nanomaterials with random‐coiled or structured DNA imparts certain stereoselectivity.^[^ ^102^ ^]^ Experimental results demonstrated that nanoparticles capped with random‐coiled DNA preferentially catalyze the oxidation of L‐glucose, while those capped with structured DNA exhibit higher activity toward D‐glucose. In cases where chiral materials are directly modified on the surface of nanomaterials, the chirality on the nanomaterials’ surface influences the orientation and structure of protein adsorption.^[^ ^103^ ^]^ Sen et al. found that AuNPs modified by different chiral glutamates greatly influence the adsorption kinetics of human serum albumin (HSA) and impact the fibrillation of adsorbed HSA.^[^ ^104^ ^]^

Furthermore, the impact of stereoisomerism extends beyond protein interactions and also affects the interaction between nanomaterials and cells. Deng et al. observed that due to the stereoisomerism between L‐phospholipid‐based cell membranes and D‐enantiomeric AuNPs, the D type is more prone to cellular identification and endocytosis.^[^ ^105^ ^]^ Studying the stereoselectivity within nano‐bio‐interfaces holds significance as it can be utilized to design biosensors for chiral molecules or develop new chiral catalytic devices.

#### 5.2.5 Hydrogen Bond Interaction

Hydrogen bond interactions are highly prevalent in biological systems and can have an impact on the interactions between nano‐bio‐interfaces (Figure 7E). Additionally, hydrogen bonds play a vital role in stereoselective interactions. In the study by Lin et al. where they examined the orientation of proteins on the surface of citric acid or MTAB‐modified AuNPs,^[^ ^106^ ^]^ it was observed that electrostatic interactions serve as the primary force for protein adsorption on the AuNP surface. However, even when the charges of AuNPs modified by different materials were the same, variations in protein adsorption were still observed. This discrepancy was attributed to the amino head of MTAB lacking protons or electron arcs, making it challenging to form hydrogen bonds and enhance the affinity between proteins and the surface. Tang et al. discovered a close relationship between the generation of stereoselectivity and hydrogen bonds.^[^ ^107^ ^]^ They found that the different arrangements of DNA on the surface of chiral‐modified nanoparticles were primarily due to the selective hydrogen bonds formed between the chiral groups and the surface of DNA molecules. While hydrogen bonding may not be the predominant factor, it remains crucial for accurately studying the nano‐bio‐interface and its interactions.

### 5.3 Chemical Reactions of ROS at Nano‐bio‐interface

ROS species are highly reactive molecules that play a dual role in biological systems. While they are necessary for various cellular processes, excessive ROS can lead to oxidative damage. Understanding the generation and elimination of ROS at the nano‐bio‐interface is essential for maintaining a balanced level of ROS. Additionally, the influence of defects in NMNs at this interface on ROS dynamics is an important area of study. By exploring the generation and elimination of ROS at the nano‐bio‐interface and considering the impact of NMN defects, we can gain insights into how to regulate ROS levels effectively and ensure optimal biological function.

#### 5.3.1 Generation and Elimination of Reactive Oxygen Species

ROS species are incompletely reduced forms of oxygen, including hydroxyl radicals, singlet oxygen, superoxide, and hydrogen peroxide.^[^ ^108^ ^]^ ROS are highly oxidizing and excessive production can lead to damage in protein and DNA structures. However, ROS also play an essential role in biological systems as information messengers, highlighting the importance of maintaining a dynamic equilibrium of ROS in organisms.^[^ ^109^ ^]^ As research on NMNs and their applications in biomedical science progresses, it has been discovered that NMNs can influence the levels of ROS in organisms. Understanding the mechanisms by which NMNs affect ROS is crucial for the safe and extensive use of NMNs. NMNs can modulate ROS levels through three primary mechanisms: 1) generating or scavenging ROS by reacting with neutral molecules such as O_2_, H_2_O_2_, and H_2_O; 2) inhibiting the scavenging of ROS through interacting with antioxidants; and 3) regulating the activity and abundance of enzymes involved in ROS generation or scavenging.^[^ ^71^ ^]^

##### Reaction with Neutral Molecules to Generate or Scavenge ROS

The ability of NMNs to generate or scavenge ROS is related to their capacity for transferring electrons between intracellular soluble species. Three main electron transfer modes exist between nano‐bio‐interfaces: direct electron injection, electron transfer mediated by surface sorbate (e.g., O_2_, H_2_O, proteins, and photosensitizers), and photogenerated electron transfer.

Studies have shown that certain NMNs, such as gold, silver, platinum and ruthenium, have the ability to scavenge ROS through direct electron injection. However, if the energetic states are not aligned, the electron‐transfer process requires the assistance of surface sorbate. For example, Palladium nanocrystals act as catalase/superoxide dismutase and remove hydrogen peroxide or superoxide by electron transfer in the presence of hydroxyl/hydrogen protons adsorbed on the crystals. Additionally, electron‐transfer can be achieved by injecting additional energy, such as light. AuNPs, for instance, can transfer electrons to the environment to generate ROS under X‐ray and UV irradiation. Understanding the generation and elimination of ROS not only paves the way for photodynamic therapy but also provides a guarantee for the safer application of NMNs.

**Figure 8:**
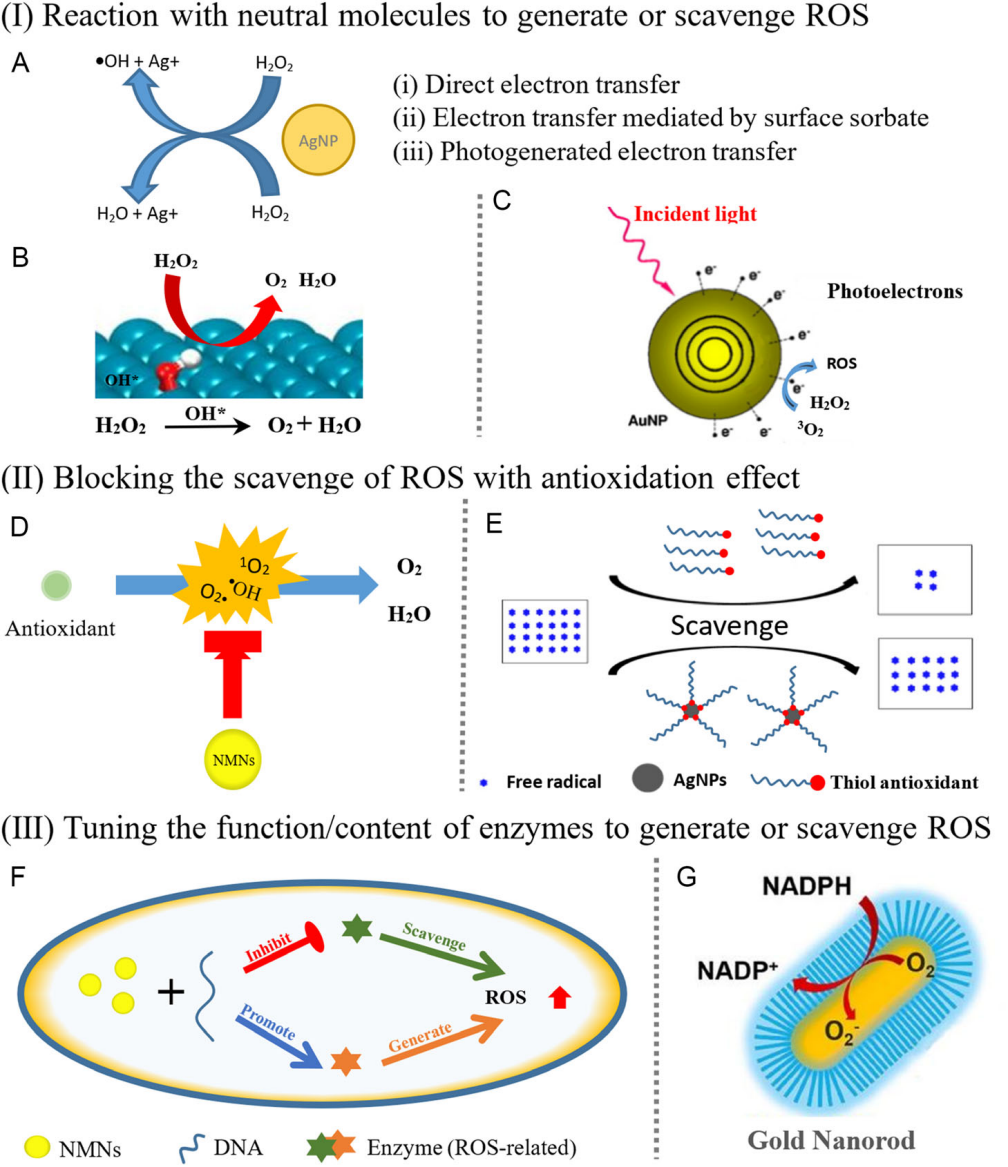
Generation and elimination of ROS. (I) Reaction with neutral molecules to generate or scavenge ROS: (A) direct electron transfer; Adapted with permission.^[^ ^111^ ^]^ Copyright 2012 Elsevier Ltd (B) electron transfer mediated by surface sorbate; Adapted with permission.^[^ ^113^ ^]^ Copyright 2016, American Chemical Society. (C) Photogenerated electronic transfer. Adapted with permission.^[^ ^114^ ^]^ Copyright 2011 Elsevier Inc. (II) Blocking the scavenge of ROS with antioxidation effect. (D,E) NMNs interacting with antioxidants to block antioxidant activity. Adapted with permission.^[^ ^115^ ^]^ Copyright 2013 American Chemical Society.(III) Tuning the function and the content of enzymes to generate or scavenge ROS: (F) NMNs affecting the expression of antioxidant related genes; (G) NMNs interacting with coenzymes. Adapted with permission.^[^ ^119^ ^]^ Copyright 2017 American Chemical Society. Adapted with permission.^[^ ^118^ ^]^ Copyright 2020 American Chemical Society.

Direct electrons can only be transferred between energetic states in the NMNs and the intracellular species that are at approximately the same energy level.^[^ ^110^ ^]^ Under acidic conditions, AgNPs demonstrated the ability to generate hydroxyl radicals by directly transferring electrons to hydrogen peroxide, referred to as the Fenton‐like effect (**Figure** **8**A).^[^ ^111^ ^]^ Previous studies have also indicated that NMNs, including gold, silver, platinum, and ruthenium, can scavenge ROS through direct electron injection.^[^ ^112^ ^]^ However, if the energetic states of NMNs and intracellular species differ, the electron transfer process necessitates the assistance of surface sorbate.^[^ ^108^ ^]^ In their investigation, Ge et al. discovered that palladium nanocrystals act as catalase/superoxide dismutase and are capable of removing hydrogen peroxide or superoxide through electron transfer in the nano‐bio‐interface by utilizing hydroxyl/hydrogen protons adsorbed on the Pd nanocrystals (Figure 8B).^[^ ^113^ ^]^ Moreover, electron transfer can be facilitated by introducing additional energy, such as light. Misawa et al. observed that AuNPs can transfer electrons to the surrounding environment, thereby generating ROS under X‐ray and UV irradiation (Figure 8C).^[^ ^114^ ^]^ The study of ROS generation and elimination not only contributes to the advancement of photodynamic therapy but also ensures the safer application of NMNs.

##### Blocking the Scavenge of ROS Through Interacting with Antioxidants

ROS play a critical role in maintaining normal cellular metabolism, but excessive ROS levels can lead to irreversible damage to cellular components. To counteract this, organisms have developed their own mechanisms to regulate ROS levels. For instance, when the concentration of ROS in cells becomes too high, antioxidants come into action to scavenge excess ROS and protect cells from oxidative damage. However, certain studies have discovered that some NMNs can interact with antioxidants like glutathione and ascorbate, indirectly causing an increase in ROS levels. In vitro experiments conducted by Zhou et al. revealed that the addition of nano‐silver significantly reduced the ability of glutathione to scavenge active free radicals. This reduction was attributed to the formation of silver‐sulfur covalent bonds between the sulfur groups of glutathione and AgNPs, which hindered its antioxidant effect (Figure 8D–E).^[^ ^115^ ^]^ Additionally, the same research team found that palladium nanomaterials can inhibit the antioxidant function of vitamin C due to the activity of ascorbate oxidation enzymes.^[^ ^116^ ^]^ This phenomenon arises from the interaction between noble metals and antioxidants, although it can be mitigated through prior modification of similar substances on the surface of nanomaterials.

##### Tuning the Function and Content of Enzymes to Generate or Scavenge ROS

As mentioned earlier, NMNs can form metal covalent bonds with antioxidants, leading to hindered antioxidation. However, in vivo experiments have revealed a different mechanism altogether. Piao et al. discovered that the addition of AgNPs also decreases the synthesis of glutamate cysteine ligase (γ‐GCL) and GSH synthetase (GSS), which are essential for glutathione synthesis.^[^ ^117^ ^]^ Additionally, NMNs can react with coenzyme NADPH in a similar fashion.(Figure 8F–G).^[^ ^118^ ^]^ Zheng and colleagues revealed that after gold nanoclusters were treated with S. aureus, the expression levels of gene dmpI and gene ilvC increased and decreased, respectively. The dmpI gene encodes enzymes that decompose benzene derivatives into intermediates of the tricarboxylic acid cycle and generate ROS. Consequently, the change in the expression level of these two genes leads to imbalances in ROS levels.^[^ ^119^ ^]^

#### 5.3.2 Influence by Defects of NMNs

Defects in nanomaterials can alter their electron‐transfer ability, consequently impacting the physicochemical properties of the materials, including their ROS‐producing ability. However, synthesizing defect‐free nanomaterials is challenging, regardless of the methods used (vapor deposition, electrodeposition, top‐down approach, or bottom‐up processes), as the types of substrates, surrounding environment, and operational modes inevitably introduce certain degrees of defects.^[^ ^120^ ^]^ The presence of crystal imperfections modifies the band gap energy (*E*_g_) of nanomaterials, facilitating easier electron transfer. As mentioned earlier, there exists a strong correlation between electron transfer intensity and ROS levels. Currently, there are numerous contradictions in the field of nanomedicine. For instance, some researchers attribute the toxicity of silver nanomaterials to the dissolution and release of silver ions.^[^ ^121^ ^]^ while others highlight the damage caused by ROS generated by silver nanomaterials.^[^ ^122^ ^]^ Scholars believe that these contradictions arise due to variations in nanomaterial synthesis methods, operational conditions, and introduced defects, which impact the physicochemical properties of the materials to varying degrees.^[^ ^120^ ^]^ Therefore, investigating nanomaterial defects holds considerable significance in understanding their intrinsic physicochemical properties.

Different shapes of nanomaterials exhibit distinct biological toxicities, which correlate with the extent of defects present. George et al. discovered that defects in silver nanomaterials accelerate their biological toxicity.^[^ ^123^ ^]^ Specifically, compared to Ag nanospheres and Ag nanowires, nanoplates exhibit a higher level of crystal defects (stacking errors and point defects). Interestingly, these defects had minimal influence on silver ion dissolution and release, but significantly enhanced the ROS‐producing ability of the silver nanomaterials.^[^ ^124^ ^]^ Notably, strong electron transfer at defects alone can effectively eliminate bacteria without generating active oxygen or causing membrane damage.^[^ ^125^ ^]^

With a deeper understanding of how defects can modify the properties of nanomaterials, researchers have recently focused on intentionally introducing defects through doping and other methods to enhance the physicochemical properties of nanomaterials, enabling them to achieve desired functionalities. For example, Qiao et al. synthesized AuZn alloy nanoclusters by doping gold with zinc, and in vitro experiments demonstrated that these nanoclusters could enhance cancer therapy by generating ROS. Additionally, zinc doping altered the electron state in the valence band of the material, leading to enhanced visible light transitions and a five‐fold increase in fluorescence intensity.^[^ ^126^ ^]^ Wongkamhaeng et al. found that the antimicrobial efficacy of platinum‐doped AgNPs increased with higher platinum doping levels, surpassing that of undoped silver or platinum nanoparticles, paving the way for potent antibacterial materials.^[^ ^127^ ^]^ Through a better understanding of nano‐defects, we can exert more precise control over the chemical properties of nanomaterials, minimize environmental effects, and maximize their practical benefits.

### 5.4 Antibacterial Mechanisms at Nano‐bio‐interface

Bacterial infections have always posed a significant threat to human health, particularly with the overuse of antibiotics leading to increased bacterial resistance. Hence, researchers urgently require alternative methods to combat bacterial threats. Recent progress in nanotechnology offers a new approach for combating such threats.^[^ ^128^ ^]^ Nanomaterials, with their excellent biocompatibility and photothermal properties, are promising candidates for use as antimicrobial agents. These materials eliminate bacterial infections mainly through four mechanisms: physical contact, oxidative stress, photothermal effect, and metal ions.^[^ ^128^ ^]^ Although each approach has its advantages and limitations, the actual application of nanomaterial sterilization typically involves multiple mechanisms.

#### 5.4.1 Physical Contact

**Figure 9:**
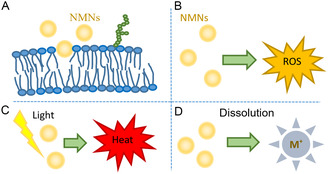
Antibacterial mechanisms of noble metal nanomaterials: (A) physical contact, (B) oxidative stress, (C) photothermal effect, and (D) release of metal ions.

Physical contact sterilization refers to the interaction between nanomaterials (NMNs) and biological molecules within bacteria, leading to the disruption of their original biological functions (**Figure** **9**A). Zhao et al. investigated the antimicrobial effect of gold nanomaterials modified with amino‐substituted pyrimidine.^[^ ^129^ ^]^ These modified gold nanomaterials can disrupt the integrity of cell membranes by chelating essential magnesium ions, which protect the structure of ribosomes and maintain membrane permeability. Furthermore, gold nanomaterials can interact with subcellular structures and bacterial DNA, thereby affecting metabolic activities such as protein production, ultimately resulting in bacterial death. It is widely accepted that positively charged silver nanoparticles (AgNPs) are adsorbed onto the cell membrane via electrostatic interactions and subsequently penetrate the interior of bacteria.^[^ ^121^ ^]^ This penetration process induces changes in the membrane structure, altering its permeability and depleting the proton power source, ultimately leading to bacterial death.

#### 5.4.2 Oxidative Stress

Oxidative stress primarily occurs due to an imbalance between oxidants and antioxidants, leading to changes in cellular structure and disruption of metabolism (Figure 9B). As mentioned earlier, NMNs can influence the levels of ROS through four different mechanisms. This unique property of noble metals allows for the manipulation of ROS levels, ultimately achieving sterilization objectives. Zheng et al. conducted a study involving a hybrid system comprising silver nanoclusters and daptomycin conjugation.^[^ ^122^ ^]^ In this study, daptomycin primarily targeted the integrity of the cell membrane, while ROS generated by silver nanoclusters contributed to the oxidation of the bacterial lipid bilayer, further exacerbating the destruction of the bacterial membrane structure. Additionally, ROS can induce DNA damage and ultimately lead to bacterial death. Rossi et al. modified gold nanomaterials on polymer films to achieve efficient light‐induced ROS sterilization.^[^ ^130^ ^]^

#### 5.4.3 Photothermal Effect

Photothermal effect refers to the localized high temperature generated by photothermal materials upon exposure to visible or near‐infrared light, which leads to sterilization (Figure 9C). Zhu et al. employed confined convective arraying techniques to design an array of gold nanorods.^[^ ^131^ ^]^ When subjected to near‐infrared light irradiation, 98% of E. coli bacteria were eradicated due to irreversible membrane damage caused by the photothermal effect. Loeb et al. synthesized a composite material consisting of gold nanorods and carbon black, possessing a wide range of light absorption.^[^ ^132^ ^]^ This composite material demonstrated effective sterilization capabilities when exposed to solar radiation. While NMNs hold great potential in photothermal therapy, several challenges must be addressed prior to their clinical application, such as understanding the response of biomolecules, including proteins, to NMNs.

#### 5.4.4 Release of Metal Ions

During the sterilization process, precious metal nanomaterials release corresponding ions to assist in the sterilization process (Figure 9D). For example, silver nanomaterials can generate silver ions through a Fenton‐like effect under acidic conditions, making silver a representative element for metal ion‐based sterilization.^[^ ^111^ ^]^ Several studies have demonstrated that silver ions affect bacterial metabolism in a similar manner to silver nanomaterials.^[^ ^133^ ^]^ Both silver nanomaterials and silver ions can induce changes in gene expression and ROS levels. Silver ions can interact with the disulfide bonds of enzymes involved in cellular metabolism, particularly respiratory enzymes. Additionally, silver ions can bind to 30 s ribosomal subunits, impeding protein synthesis.^[^ ^121^ ^]^ It is important to note that the dissociation of nanomaterials and the presence of ions only contribute partially to the germicidal effect, serving as an auxiliary method to achieve sterilization.

### 5.5 Anticancer Mechanisms at Nano‐bio‐interface

Cancer is a major disease that poses a significant threat to human health. Current treatment options for cancer include surgery, chemotherapy, and radiotherapy. However, these methods have limitations such as the incomplete eradication and the potential for relapse, as well as damage to normal cells, which can increase the patient's physical burden.^[^ ^134^ ^]^ With the rapid development of nanomaterials in the field of biomedicine, researchers have discovered new ways to treat cancer. In particular, noble metal nano‐bio‐interfaces have shown promise in the anticancer process.

There are three main ways in which noble metal nanoparticles can exert an anticancer effect: photothermal anticancer, photodynamic anticancer, and immunotherapy.^[^ ^135^ ^]^ Photothermal and photodynamic anticancer mechanisms are similar to the antibacterial mechanism of NMNs, achieved through the excellent photothermal conversion ability or photocatalytic ability of NMNs. However, due to the complexity of biological environments, NMNs interact with various biomolecules or cells to produce various nano‐bio‐interfaces, which determine their ultimate fate in living organisms. By adjusting the surface morphology, surface charge, and surface coating of NMNs, researchers can control the formation of nano‐bio‐interfaces and the fate of the nanomaterials. Furthermore, the nano‐bio‐interface can be adjusted through external stimulation to enhance the effectiveness of photothermal treatment. For instance, Park et al. designed a pH‐responsive AuNP by modifying ssDNA and cytochrome c on its surface. Under acidic conditions, the AuNPs aggregated due to electrostatic interactions, leading to a red shift in the absorption spectrum of the aggregated particles and thereby achieving improved photothermal conversion efficiency.^[^ ^136^ ^]^ This pH effect is particularly advantageous for tumor treatment, given that the microenvironment of tumor cells tends to be slightly acidic compared to normal cells.

NMNs also play a crucial role in immunotherapy due to their biological and optical stability. Immunological drugs or immune‐related regulatory proteins can be modified on the surface of precious metal nanomaterials, which are then internalized by cancer cells to achieve the goal of immunotherapy. The nano‐bio‐interface ensures that the loaded nano‐drugs can enter the cancer cells in immunotherapy, but it is more than that. External and internal stimuli can change the nano‐bio‐interface to achieve targeted treatment of cancer. For example, heat‐sensitive components can be inserted between AuNPs and immune drugs, and external light stimulation can trigger the photothermal conversion ability of the AuNPs to release immune drugs.^[^ ^137^ ^]^ The slightly acidic microenvironment of tumor cells can also be leveraged to achieve targeted treatment.^[^ ^138^ ^]^

## 6 Technical Probing of NMNs at Nano‐bio‐interface

Revealing the underlying interactions of NMNs with biomolecules, cells and tissues at the nano‐bio‐interface is crucial for designing and optimizing their structure, properties, uptake, distribution, metabolism, and degradation in both in vivo and in vitro settings. However, our current understanding of the structure and biophysical and chemical interactions at the nano‐bio‐interface is limited. Revealing the mechanisms of NMN‐biomolecule interactions is challenging due to the complex and dynamic nature of the nano‐bio‐interface. Therefore, there is a need for analytical methods and techniques to study these interactions. A variety of methods have been used to explore the NMNs nano‐bio‐interface, including microscopy imaging techniques, spectroscopy methods, X‐ray based methodology, time‐resolved microscopy, and other tools. However, in‐depth investigations are still needed to fully comprehend the nano‐bio‐interface and the interactions that occur within it.

### 6.1 Microscopic Imaging

Electron microscopy techniques such as transmission electron microscopy (TEM) and SEM provide valuable information regarding the size and morphology of the interactions between NMNs and biomolecules. AFM is capable of detecting subnanometer‐scale phenomena and measuring the force between NMNs and biomolecules, tissues, or cell surfaces, which indicates the binding strength. In a study by Shan et al. applied force spectroscopy based on AFM was employed to investigate the interaction between quantum dots and living cells.^[^ ^139^ ^]^ The quantum dots with a diameter of ≈4 nm were attached to the tip of a miniature AFM to measure the force exerted by a single quantum dot interacting with living HeLa cells under natural conditions. Before conducting force spectroscopy, a charge‐coupled device (CCD) camera was used to locate the AFM tip precisely on top of a HeLa cell monolayer. With the AFM cantilever positioned above the monolayer, successful force spectroscopy measurement was ensured. Using this technique, l‐cysteine stabilized gold nanoparticles (AuNPs) were covalently conjugated to an AFM tip via a heterobifunctional aldehyde‐PEG (poly(ethylene glycol))‐NHS linker. The interaction force between a single AuNP and a HeLa cell under physiological conditions was measured, demonstrating that the interaction force increased with the size of the AuNPs and was dependent on the surface charges of the AuNPs.

Shan et al. observed cellular interactions of gold nanoclusters (AuNCs) using various techniques including confocal fluorescence microscopy, scanning transmission electron microscopy,^[^ ^140^ ^]^ inductively coupled plasma optical emission spectroscopy, and cell viability assays. They found that the adherence to cell membranes, uptake efficiency, and cytotoxicity greatly depended on the surface charges of human serum albumin (HSA) variants adsorbed onto the AuNPs. These findings indicated that cellular responses to nanoparticle exposure rely on specific properties of the proteins that adsorb onto nanoparticles from biofluids.

### 6.2 Spectroscopic Measurement/Examination

Optical spectroscopy is a commonly used method to study the interaction between nanoparticles and biomolecules, mainly including UV‐Vis absorption, fluorescence, Raman, circular dichroism, and infrared spectroscopy. Spectroscopic methods play an important role in studying binding affinity, binding ratio, and binding mechanisms. Fluorescence spectroscopy is particularly sensitive to protein dynamics because the excited fluorescence state lasts for nanoseconds, which coincides with many important biological processes such as molecular binding and protein conformational changes. Steady‐state fluorescence spectroscopy, fluorescence resonance energy transfer, or stepwise monomolecular photobleaching can be used to monitor NMNs‐protein binding.

Garabagiu et al. applied UV‐Vis and fluorescence spectroscopy to study the interaction process between horse hemoglobin and AuNPs.^[^ ^141^ ^]^ In the presence of AuNPs, the Soret band of hemoglobin did not show significant changes, indicating that the protein retained its biological function. The binding ratio of protein on NMNs could be determined using fluorescence polarization. Wang et al. studied the conformational change of papain induced by AuNPs using UV‐Vis absorption, fluorescence, and synchronous fluorescence spectroscopic techniques under physiological conditions.^[^ ^142^ ^]^ The study showed that hydrogen bonding and Van der Waals forces play a key role in the interaction according to the calculated thermodynamic parameters (Δ*H*, Δ*S*, and Δ*G*). These strategies help to understand the transportation and distribution of NMNs in biological systems and develop the application of NMNs in biomedicine. However, these studies generally focus on the interaction between a specific protein with NMNs or provide limited information on the overall condition of the protein corona, limiting further understanding of nano‐bio‐interactions in practical applications.

Circular dichroism (CD) spectroscopy is a powerful tool for studying the stability and changes in the structure of biomolecules bound to NMNs. It utilizes characteristic CD spectra in the UV region to analyze these structural properties. Rahmani et al. used CD spectroscopy and fluorescence spectroscopy to investigate the interaction between AgNPs and tau protein, as well as the SH‐SY5Y neuroblastoma cell line.^[^ ^143^ ^]^ Their fluorescence study revealed that AgNPs with a diameter of 10–20 nm form a static complex with tau protein through hydrogen bonds and Van der Waals interactions. CD experiments showed that AgNPs did not affect the random coil structure of tau protein. Similarly, Shang et al. employed fluorescence and CD spectroscopy to study structural changes and binding affinities in HSA variants.^[^ ^144^ ^]^ However, CD spectroscopy is limited in its application to complex protein mixtures, and, like other spectroscopic methods, it provides an average representation of the molecular population.

### 6.3 Synchrotron Radiation‐based Analytics

The use of X‐ray‐based analytics, such as X‐ray fluorescence, diffraction, refractive imaging methods, and synchrotron radiation (SR)‐based analysis techniques, can reveal the distribution and translocation of unlabelled NMNs in biological samples, detect different components in nanoparticle‐based drugs, and visualize NMNs at cellular and subcellular interfaces. Wang et al. provided a comprehensive overview of SR‐based techniques and their advantages, such as simple sample handling, spatial resolution as low as 10 nm, and penetration depth of tens of millimeters.^[^ ^145^ ^]^ These techniques can distinguish objects 5–10 times smaller than traditional light microscopes and reveal site‐specific interactions at the nano‐bio‐interface. Wang et al. used a multi‐analytical approach based on SR‐based X‐ray absorption spectroscopy (SR‐XAS), microbeam X‐ray fluorescence (SR‐μXRF), and circular dichroism (SR‐CD) spectroscopy to identify at least 12 Au—S bonds linking the corona on the gold surface of CTAB‐coated Au nanorods and characterized their binding modes.^[^ ^146^ ^]^ They used SR‐TXM and SR‐XANES spectroscopy to capture the 3D distribution of AgNPs in living cells and found that cytotoxicity was largely caused by the chemical transformation of particulate silver from Ag^0^ to Ag^+^ and Ag—O^−^, then Ag—S^−^ species. They also have combined SR‐based transmission X‐ray microscopy (SR‐TXM) and SR‐based X‐ray absorption near edge structure (SR‐XANES) spectroscopy to capture the 3D distribution of AgNPs in living cells and found that cytotoxicity was largely caused by the chemical transformation of particulate silver from Ag^0^ to Ag^+^ and Ag—O^−^, then Ag—S^−^ species.^[^ ^147^ ^]^

### 6.4 Time‐scale Observations

The time‐scale observation at nano‐bio‐interface is of great significance for understanding the dynamic changes of the NMNs nano‐bio‐interface. In recent years, high‐temporal resolution and ultra‐fast time resolution techniques have aroused the interest of a wide range of researchers. Time‐resolved ultrafast spectroscopy can provide more detailed information that is not observable by steady‐state spectroscopy. At present, the time resolution capability of ultrafast spectroscopy technology has reached the order of picoseconds and femtoseconds, providing a powerful tool for observing the ultrafast process of interaction between NMNs and biomolecules. With this technology, it is possible to obtain information about the rapid changes in the internal molecular state of the ultrafast process, and to further understand the mechanism and dynamics of the microscopic process, such as stroke, energy transfer, charge transfer, and molecular orientation of the intermediate state. In recent years, time‐correlated single photon counting (TCSPC) measurement has been used as a powerful tool to reveal the time evolution of fluorescence intensity and lifetime during hybridization. It has the advantages of ultra‐high resolution (6 ps), ultra‐high sensitivity (less than a single photon level), short measurement time, high dynamic range, high linearity, and excellent signal‐to‐noise ratio. This technology is an important means to understand and develop NMNs nano‐bio‐interface. Wei et al. applied time‐resolved fluorescence spectroscopy based on TCSPC technique to investigate the conformation states of hairpin DNA on the surface of AuNPs and energy transfer processes in Au‐nanobeacons.^[^ ^148^ ^]^ TCSPC has opened promising possibilities for real‐time monitoring of hybridization events using Au‐nanobeacons.

### 6.5 More Interfacial Analysis/Examination

Besides, a number of other technologies have also been used to resolve nano‐bio‐interactions. For instance, the zeta potential provides information about the nature of the surface charge of the NMNs. It can be used to detect the binding of biomolecules to the NMNs surface, because this will change the overall surface charge. Surface plasmon resonance (SPR) is a label‐free method that can measure the changes in optical parameters of the nano‐bio‐interactions based on the changes in surface plasmon wave oscillation caused by the adsorption of biomolecules on the metal surface. This approach can reliably analyze the selectivity, binding affinity and binding kinetics between biomolecules and NMNs without any prerequisites, such as sample fluorescence, absorbance or enthalpy changes. Dynamic light scattering (DLS) is used to determine small changes in the hydrodynamic radii of noble metal nanoparticles in solution and to detect the size distribution. Binding of proteins or other biomolecules causes an increase in the dynamic radius of nanoparticles and this increase will stop when the binding is saturated. This can be used to monitor the binding ratio. Stetefeld et al. provided examples of DLS's application for homogeneity and interaction studies of proteins, nucleic acids, and their complexes.^[^ ^149^ ^]^ However, it is not easy to characterize heterogeneous biological samples using DLS, as it requires multi‐angle detection combined with experimental knowledge to interpret the data. All the nanoparticles present in a biological sample, such as proteins and other bio‐matter, contribute to the scattered light and obscure the information about the colloidal NMNs. The scattering intensity is proportional to the sixth power of particle radius (*R*_H_^6^), and the agglomeration phenomenon caused by nanoparticles instability in biological environment may be dominant.

In general, single method is limited, and multiple techniques need to be utilized comprehensively to study different aspects of the NMNs nano‐bio‐interface. Placido et al. monitored the bioconjugation procedure by the combined analysis of UV‐Vis absorption, resonance Raman and Fourier transform infrared spectroscopy, transmission electron microscopy, and ζ‐potential, verifying the successful conjugation of the horse heart cytochrome c protein to the Au nanorods.^[^ ^150^ ^]^ The thorough characterization of the bioconjugation procedure established that the native state of the protein has been retained upon conjugation with Au nanorods and that the plasmonic properties of the Au nanorods have also been mostly maintained.

## 7 Toxicity and Cellular Internalization of NMNs at Nano‐bio‐interface

Influencing factors on toxicity and cellular internalization of NMNs at nano‐bio‐interface

Nanomaterials have been involved into almost every aspects of our life in our current era. Subsequently, the concern about the toxicity of nanomaterials has been got more and more attention. NMNs have been widely used because of their ease of synthesis, characterization, surface functionalization and size/shape‐dependent optoelectronic properties. Compared with other nanomaterials, NMNs have better biocompatibility except silver nanomaterials, cellular internalization of NMNs will not significantly affect the normal metabolic activity of cells. Therefore, this part not only discusses the factors that affect the toxicity of NMNs but also discusses the factors that affect the internalization of NMNs. The reason that we discuss the cellular internalization behavior of NMNs is that we have reasons to think that the internalization of NMNs is the first step of the cytotoxicity caused by NMNs. Nanoscale materials are more susceptible to various factors, such as the size, shape, surface charge and surface coating of NMNs. Investigation the influence of various factors on the toxicity of nanomaterials is helpful for researchers to design NMNs with low toxicity and realize the safer application of NMNs. The following are the effects of size, shape, surface charge and surface coating on the toxicity and cellular internalization of NMNs.

### 7.1 Particle Size

**Figure 10:**
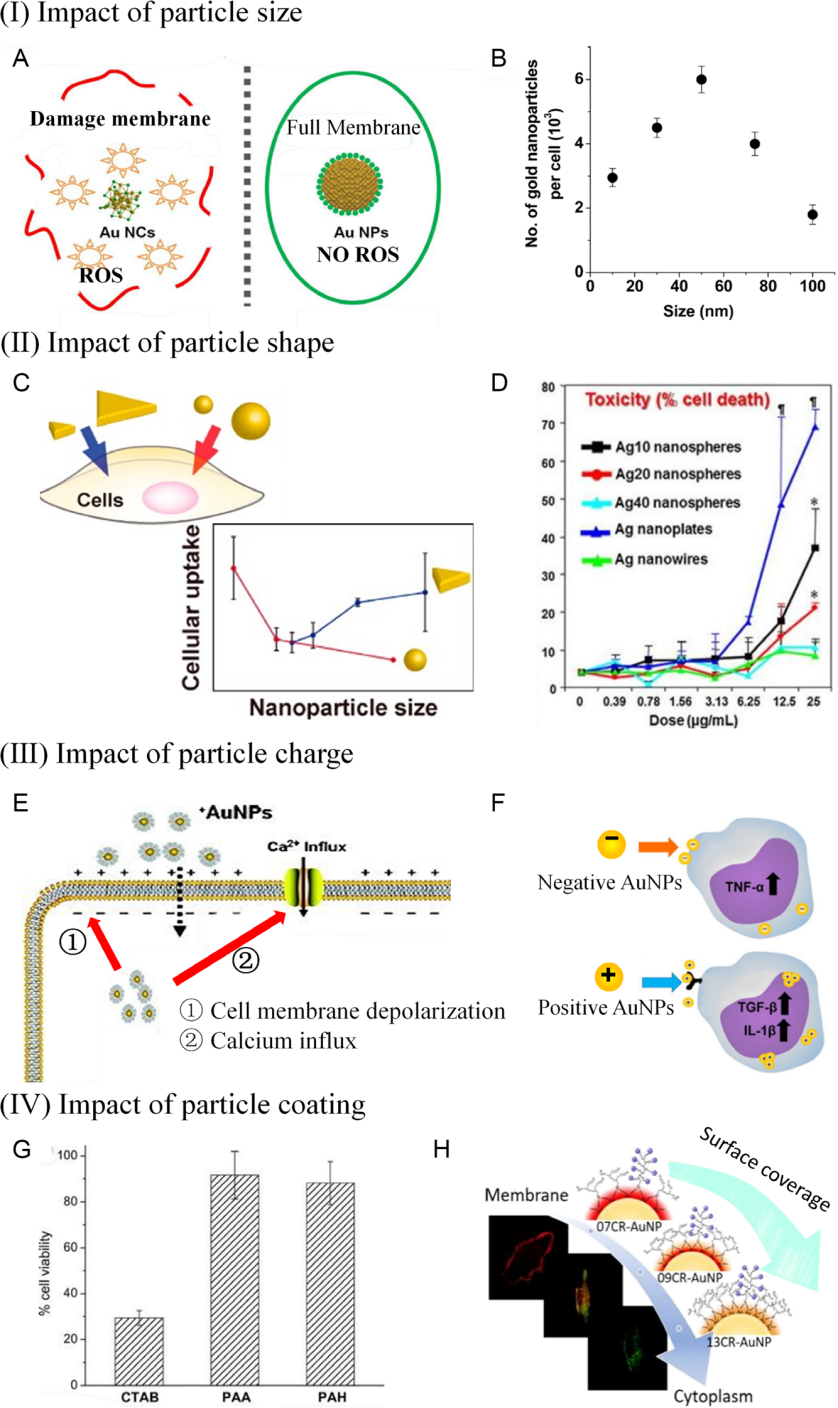
Factors influencing the toxicity and cellular internalization of NMNs. (I) Impact of particle size: (A) relationship between size and catalytic activity of NMNs; Adapted with permission.^[^ ^119^ ^]^ Copyright 2017, American Chemical Society. (B) relationship between size and cell internalization. Adapted with permission.^[^ ^153^ ^]^ Copyright 2006, American Chemical Society. (II) Impact of particle shape: (C) relationship between shape and cell internalization; Adapted with permission.^[^ ^154^ ^]^ Copyright 2016, American Chemical Society. (D) Relationship between shape and cytotoxicity. Adapted with permission.^[^ ^123^ ^]^ Copyright 2012, American Chemical Society. (III) Impact of particle charge: (E, F) charge on the surface of NMNs affecting the metabolic activities of cells. Adapted with permission.^[^ ^157^ ^]^ Copyright 2010, American Chemical Society. Adapted with permission.^[^ ^158^ ^]^ Copyright 2020, American Chemical Society.(IV) Impact of particle coating: (G) relationship between surface coverage and cell internalization; Adapted with permission.^[^ ^159^ ^]^ Copyright 2009, Wiley‐VCH. (H) Relationship between types of surface coating materials and cytotoxicity. Adapted with permission.^[^ ^160^ ^]^ Copyright 2019, American Chemical Society.

It is well known that smaller nanomaterials lead to stronger cytotoxicity. The main reason for this phenomenon is mainly related to the following two points: 1) smaller NMNs possess more catalytic activity; 2) cellular internalization of NMNs have size selectivity. Freitas et al. explored the toxicity of silver nanomaterials at different scales to human neutrophils by using various methods such as Trypan blue exclusion method, propidium iodide staining method and Neutral red uptake method.^[^ ^151^ ^]^ It was found that the smaller silver nanomaterials are more likely to lead to necrocytosis, in addition, by using transmission electron microscopy for nanomaterials cell distribution in different size, 10 nm AgNPs are widely distributed in the cytosol, but most of the large AgNPs are blocked outside the cell by the cell membrane and cannot enter the cell. Zheng et al. found that gold nanoclusters have higher catalytic activity than AuNPs,^[^ ^119^ ^]^ which can induce more ROS in cells, resulting in more serious destruction of important components of cells (**Figure** **10**A). Onodera et al. also investigated the size‐dependent cytotoxicity of AgNPs.^[^ ^152^ ^]^ It was found that smaller AgNPs can induce higher levels of mitochondrial ROS, which can induce apoptosis by promoting intrinsic apoptosis pathway. However, the experiments did not specifically measure the ability of silver nanomaterials of different sizes to enter cells. In fact, few studies have combined the two perspectives to study the effect of the size on cytotoxicity. Chithrani et al. previously studied the influence of the size of nanomaterials on cell internalization.^[^ ^153^ ^]^ In the experiment, gold nanospheres of different sizes from 14 to 100 nm were designed respectively to investigate the difference of cell internalization (Figure 10B). The experimental results found that medium‐sized AuNPs enter cells more efficiently through receptor‐mediated endocytosis than smaller nanoparticles. The results of the above experiments support the view that the effects of size factors on biological toxicity is the result of the combined action of these two perspectives.

### 7.2 Particle Shape

The shape of nanomaterials also has a certain influence on biotoxicity, the reasons can be divided into two aspects: 1) cells have a shape selectivity of the internalization of NMNs; 2) different shapes of NMNs have different catalytic activities. Ijiro et al. studied the effect of the shape of nanomaterials on cell internalization,^[^ ^154^ ^]^ and found that when the surface area was larger than 0.5 × 10^4^ nm^2^, cells (RAW264.7 and HeLa cells) showed obvious preference for the internalization of triangular gold nanomaterials (Figure 10C). The researchers believed that the edges and vertices of triangular nanomaterials with high local curvature were main reason to accelerate their internalization. Different shapes of nanomaterials have different catalytic activities. Andree. George et al. found that under the same condition, Ag nanoplates could generated more ROSthan nanospheres and nanowires, leading to severe oxidative stress.^[^ ^123^ ^]^ This shape‐dependence catalytic activities is mainly due to the fact that nanoplates is more likely to produce highly active sites (defects) than other nano shape. Besides, different shapes of NMNs have different dominant crystal structures, and the crystal structures of NMNs will have different effects on the conformation and function of biomolecules adsorb on their surfaces (Figure 10D). Gagner et al. found that the adsorption affinity of proteins on the Au{111} surface was higher than that on the Au {100} surface.^[^ ^155^ ^]^ The preliminary analysis of the researchers suggested that this phenomenon might be caused by the higher packing density of negatively charged ligands on the Au {111} surface. Similarly, Feng et al. noted that proteins are more likely to attach to relatively flat surfaces.^[^ ^156^ ^]^ Nanomaterials entering cells will adsorb biological components to different degrees due to different crystal structures, and adsorbate on the surface of nanomaterials will undergo conformational changes, thus leading to functional changes. Severe functional changes will lead to cell metabolism imbalance and cell death. There may be other mechanisms for shape‐induced cytotoxicity, but the focus of research is on whether shape affects cell internalization and whether shape affects the physicochemical properties of materials.

### 7.3 Particle Charge

The charge on the surface of nanomaterials not only stabilizes the nanoparticles but also has a certain influence on the toxicity of nanomaterials. The electronegativity of phospholipid bilayer structure of cell membrane, positively charged nanomaterials compared with negatively charged and neutral nanoparticles are more likely to adsorb on the surface of cell membrane due to the electrostatic interaction, thus more likely to enter the cytosol and affect the normal metabolism of cells. Not only cell membranes, most biomolecule (DNA, proteins, etc.) in organisms are charged, so the adsorption interaction and subsequent effects of different charged nanomaterials in organisms will also be different. Arvizo et al. found that positively charged AuNPs depolarize the membrane to the greatest extent,^[^ ^157^ ^]^ while nanoparticles of other charges having negligible effect. Such membrane potential perturbations result in increased [Ca^2+^], which in turn inhibits the proliferation of normal cells whereas malignant cells remain unaffected (Figure 10E). Srijampa et al. studied the effect of charged AuNPs on the behavior of monocytes and found that compared with anionic nanoparticles, cationic nanoparticles can enter the cytosol through the endocytosis mediated by the caveolae‐mediated endocytosis, thus increasing the content of cationic nanoparticles inside the cell. Neither cationic nor anionic nanoparticles enter the cell without damaging the integrity of the cell membrane. But there are differences in the impact of nanoparticles with different charges, for example, Cationic nanoparticles could weakly induce the expression of pro‐inflammatory (IL‐1β) anti‐inflammatory (TGF‐β) cytokines, while anionic nanoparticles could promote the expression of TNF‐α (Figure 10F).^[^ ^158^ ^]^ Few reports that the charge of the surface of the nanomaterials will affect the catalytic performance of the material itself. Surface modified substances are essential for nanomaterials as a stabilizer. In the field of surface charge on the toxicity of nanomaterials, this research area has reached a consensus: Cationic nanoparticles have relatively higher toxicity than the anionic nanoparticles.

### 7.4 Particle Coating

In order to achieve better treatment and diagnosis of nanomaterials, common ways are modified some functional molecules on the surface of nanomaterials. Functional molecules are more than just common molecules (CTAB, citric acid, etc.) having stabilizing nanomaterials, but also include certain biological macromolecules such as proteins, DNA. It has been found that the toxicity of NMNs are not only derived from noble metals themselves, but some desorption surface coatings are also toxic to the cells. Alkilany et al. support that the cytotoxicity of CTAB‐modified Au nanorods was derived from desorption CTAB,^[^ ^159^ ^]^ and that the desorption of CTAB and the cytotoxicity of nanoparticles were effectively reduced by coating the surface of CTAB with a layer of serum proteins.(Figure 10G) In addition, the cell internalization of protein‐coated nanoparticles was significantly increased. Gong et al. modified ultra‐small AuNPs with glutathione and cell‐penetrating peptide (CR8).^[^ ^160^ ^]^ The results showed that lower surface coverage leads to rapid cellular interaction and strong membrane binding, but low cellular internalization, whereas high surface coverage showed slow cellular interaction and low membrane binding, but major cellular internalization (Figure 10H). This discovery enables us to successfully regulate cell interaction and the ability to internalize nanoparticles by introducing some functional ligands on the surface of nanomaterials.

There are still many studies on the cytotoxicity of NMNs, and contradictory viewpoints emerge one after another. For example, in the effect of the size of NMNs on cell internalization, Fu et al. view that the smaller nanoparticles are more likely to internalize cells is not consistent with that of Chithrani.^[^ ^161^ ^]^ Authors consider that the differences in experimental results are caused by a variety of factors, such as the temperature of the experiment, the type of culture medium and cells. The difference of cell type may be the main reason. Hela cells used in Chithrani's internalize different nanomaterials through different endocytosis, while MCF‐7 Cells used in Fu's experiments, nanoparticles through penetration into cell. Of course, this is only a guess, the real reasons for the contradictory views need to be further explored, perhaps these contradictory views are the significance of the study.

## 8 Outlook and Perspective

Nano‐bio‐interface plays a crucial role in the biological effects of nanomaterials, and manipulating this interface can facilitate their applications. The existence of the nano‐bio‐interface presents both advantages and disadvantages for nanomaterials’ biological effects.^[^ ^162^ ^]^ When unmodified NMNs enter an organism, they interact randomly with surrounding biological components such as proteins and phagocytes. This interaction can lead to the formation of a protein crown around the nanomaterial, which then determines its biological effect instead of the nanomaterial itself. Consequently, the original role of the nanomaterial in the organism may be hindered. Furthermore, the surface of nanomaterials can adsorb unique signal proteins like opsonins, resulting in rapid excretion of the nanomaterials and adversely affecting their biological properties.^[^ ^163^ ^]^ These adverse effects stem from a lack of understanding of the interaction mechanisms at the nano‐bio‐interface.

However, further research into the nano‐bio‐interface has shown that it can actually help nanomaterials achieve better biological effects. Pre‐modifying nanomaterial surfaces with proteins, which have minimal impact on the biological effects, can form protein crowns that inhibit the adsorption of other proteins that significantly influence the biological effects. Additionally, modifying specific signal proteins, such as CD45 or CD47, on the nanomaterial surface can prolong their circulation time in vivo, preventing rapid excretion. Another approach involves modifying nanomaterials with recognition proteins that target receptor proteins on the cell membranes of specific cells, enabling targeted therapy.^[^ ^162^ ^]^ By leveraging these strategies and advancements in our understanding of the nano‐bio‐interface, we can enhance the biological effects of nanomaterials. It is important to note that the adverse effects observed thus far are due to a limited understanding of the nano‐bio‐interface. As our knowledge improves, we anticipate significant breakthroughs in the biological effects of nanomaterials.

Moreover, the simple synthesis method and unique properties of NMNs have led to their widespread use in constructing various nano‐reaction interfaces, giving birth to nano‐biotechnology and other emerging research fields. Although significant progress has been made in the development of biological applications of NMNs, this emerging field still faces many challenges. One major challenge is understanding the interaction between NMNs and biomolecules in living organisms. While adjusting the size and surface chemistry of NMNs can be used to treat diseases, the process and mode of interaction between NMNs and biomolecules is still unclear. Additionally, the behavior of various biological macromolecules on the interface of NMNs remains difficult to accurately grasp. Another challenge is predicting changes in the structure and function of proteins after interacting with NMNs, which may affect the physicochemical properties and application quality of NMNs. The quantitative relationship between the structure, physicochemical properties, and toxicity of noble metal nanoparticles is also not fully understood.

To address these challenges, it is imperative to deploy advanced technologies and equipment for dynamic, in‐situ, real‐time, ultra‐high‐speed, and high‐resolution collaborative research on the interaction process between NMNs and biomolecules. Otto et al. applied X‐ray photon correlation spectroscopy (XPCS) to observe the dynamics of AuNPs in a biological environment, enabling the –in‐situ analysis of their behavior in such a setting.^[^ ^164^ ^]^ XPCS, similar to dynamic light scattering technology, overcomes the limitations of DLS. DLS, which relies on visible light detection technology, is significantly impacted by scattering, absorption, and reflection effects when used for in‐situ detection in a biological environment. By calculating the intensity autocorrelation function and employing the diffusion model, the hydrodynamic diameter of nanomaterials can be determined and monitored, thereby providing preliminary insights into the interaction of nanomaterials in a biological environment.^[^ ^164^ ^]^ As stated, XPCS is still in its nascent stage as a technology for studying nano‐bio‐ interactions. With the advancement of fourth‐generation synchronous acceleration technology, X‐ray technology is poised to make a substantial contribution to the study of the biological behavior of nanomaterials in biological environments. Computational chemistry can be used to establish a surface state model of the interaction between NMNs and the complex environment in the body to ensure safety and effectiveness. We believe that solving these problems will promote scientific and technological advancement, especially in the fields of disease diagnosis, cancer treatment and environmental pollution. Therefore, future research should pay more attention to theoretical simulations to accurately and in‐depth study nano‐bio‐interactions, paving the way for safe and effective biomedical applications of NMNs bio‐interface.

## Conflict of Interest

The authors declare no conflict of interest.
